# Induction of Wnt-Inducible Signaling Protein-1 Correlates with Invasive Breast Cancer Oncogenesis and Reduced Type 1 Cell-Mediated Cytotoxic Immunity: A Retrospective Study

**DOI:** 10.1371/journal.pcbi.1003409

**Published:** 2014-01-09

**Authors:** David J. Klinke II

**Affiliations:** 1 Department of Chemical Engineering and Mary Babb Randolph Cancer Center, West Virginia University, Morgantown, West Virginia, United States of America; 2 Department of Microbiology, Immunology, and Cell Biology, West Virginia University, Morgantown, West Virginia, United States of America; Princeton University, United States of America

## Abstract

Innate and type 1 cell-mediated cytotoxic immunity function as important extracellular control mechanisms that maintain cellular homeostasis. Interleukin-12 (IL12) is an important cytokine that links innate immunity with type 1 cell-mediated cytotoxic immunity. We recently observed in vitro that tumor-derived Wnt-inducible signaling protein-1 (WISP1) exerts paracrine action to suppress IL12 signaling. The objective of this retrospective study was three fold: 1) to determine whether a gene signature associated with type 1 cell-mediated cytotoxic immunity was correlated with overall survival, 2) to determine whether WISP1 expression is increased in invasive breast cancer, and 3) to determine whether a gene signature consistent with inhibition of IL12 signaling correlates with WISP1 expression. Clinical information and mRNA expression for genes associated with anti-tumor immunity were obtained from the invasive breast cancer arm of the Cancer Genome Atlas study. Patient cohorts were identified using hierarchical clustering. The immune signatures associated with the patient cohorts were interpreted using model-based inference of immune polarization. Reverse phase protein array, tissue microarray, and quantitative flow cytometry in breast cancer cell lines were used to validate observed differences in gene expression. We found that type 1 cell-mediated cytotoxic immunity was correlated with increased survival in patients with invasive breast cancer, especially in patients with invasive triple negative breast cancer. Oncogenic transformation in invasive breast cancer was associated with an increase in WISP1. The gene expression signature in invasive breast cancer was consistent with WISP1 as a paracrine inhibitor of type 1 cell-mediated immunity through inhibiting IL12 signaling and promoting type 2 immunity. Moreover, model-based inference helped identify appropriate immune signatures that can be used as design constraints in genetically engineering better pre-clinical models of breast cancer.

## Author Summary

Effective anti-tumor immunity is proportional to the number and to the cytotoxic activity of immune cells that enter the tumor microenvironment. Recent advances in cancer immunotherapy stem from increasing the number of tumor-infiltrating immune cells by inhibiting immune checkpoints or adoptive T cell therapy. Here, we used computational methods to identify potential mechanisms present within the tumor microenvironment that limit the efficacy of anti-tumor immunity. Specifically, we found that oncogenic transformation is associated with the induction of tumor-derived biochemical cues, namely Wnt-inducible signaling protein-1, that locally suppress anti-tumor immunity. Moreover, we used model-based inference to demonstrate that a gene signature consistent with effective type 1 cell-mediated cytotoxic immunity is a predictor of overall survival independent of molecular pathology. Interestingly, patients with triple negative breast cancer were more enriched in the cohort associated with type 1 cell-mediated immunity. As this immune gene signature is not present in current genetically engineered mouse models of breast cancer, the results help identify design constraints for engineering better pre-clinical models of breast cancer. Demonstrating efficacy in pre-clinical animal models is a pre-requisite for bringing improved cancer immunotherapies into the clinic.

## Introduction

The discovery of molecular targeted therapies revolutionized the treatment of breast cancer. Tamoxifen, a small molecule inhibitor of the estrogen receptor, was the first drug to inhibit the growth of breast cancer cells that depend on female sex hormones. More recently, trastuzumab was developed to inhibit the growth of breast cancer cells that overexpress HER2, an oncogenic member of the epidermal growth factor family of receptors [1]. Based upon their demonstrated clinical impact, a pre-operative biopsy sample is used to guide treatment based upon expression of the hormone receptors for estrogen (ER) and progesterone (PR) and the epidermal growth factor receptor HER2 [2]. While these molecular targeted therapies have improved survival, de novo and acquired resistance to these therapies present challenges for achieving a durable clinical response [3], [4].

The difficulties in achieving a durable clinical response using molecular targeted therapies have sparked a renewed interest in viewing cancer from an evolutionary perspective [5]–[7]. Thinking about cancer from an evolutionary perspective involves three key concepts. First, tumors are comprised of a heterogenous population of cells. While non-genetic sources of heterogeneity have been recognized for several decades [8], recent studies of breast cancer using next generation sequencing have revealed the genetic heterogeneity associated with oncogenesis [9]–[14]. Second, the different cell types contained within the tumor microenvironment - stromal cells, malignant clones, and cells of the immune system - and their collective interactions comprise a dynamic system. Dynamic systems typically have control mechanism that aim to maintain the system in a desirable state, such as tissue homeostasis, in the presence of external perturbations [15]. Identifying the control architecture in biological systems remains a central challenge. Third, cells of the population impinge upon a selective fitness landscape that determines their fate. The selective landscape is composed of intracellular and extracellular adaptive control mechanisms that regulate tissue homeostasis. Many intracellular control mechanisms are well studied and form the foundation of the hallmarks of cancer [16]. Innate and adaptive immunity function as extracellular control mechanisms that regulate cellular homeostasis [17] and, in contrast, are not well understood [5].

Cytokines coordinate innate and adaptive immunity and defects in their action have pathogenic implications. For instance, Interleukin-12 (IL12) is a cytokine that is produced by innate immune cells and acts upon Natural Killer cells, CD8^+^ Cytotoxic T cells, and CD4^+^ T helper cells to initiate a type 1 cell-mediated adaptive immune response [18]. Genetic mutations in IL12p40 and one component of the IL12 receptor, IL12R*β*1, have been observed in patients with recurrent mycobacterial disease, suggestive of insufficient type 1 cell-mediated immunity [19], [20]. Genetic deletion of other component of the IL12 receptor, IL12R*β*2, increases susceptibility to spontaneous autoimmunity, B-cell malignancies, and lung carcinomas [21]. Originally called Natural Killer (NK) Cell Stimulating Factor, IL12 also enhances the ability of NK and CD8^+^ Cytotoxic T cells to lyse target cells, a mechanism exploited for tumor immunotherapy. For example, IL12 was used as an adjuvant to promote NK-cell mediated killing of HER2-positive breast cancer cells in patients treated with trastuzumab [22]. As an adjuvant for tumor immunotherapy, toxicity restricts the systemic delivery of IL12 [23]. However, local delivery of IL12 to the tumor microenvironment promotes tumor regression in the B16 melanoma model [24] and in the EL4 thymoma model [25]. Given that genetic defects in IL12 signaling increase cancer incidence and enhanced local delivery of IL12 promotes tumor regression, we recently asked whether malignant cells alter the selective fitness landscape by locally inhibiting the response of immune cells to IL12. Using the B16 model for melanoma, we identified that tumor-derived Wnt-inducible signaling protein 1 (WISP1), a beta-catenin responsive oncogene [26], exerts paracrine action on immune cells by inhibiting their functional response to IL12 [27]. The objective of this retrospective study of invasive breast cancer was three-fold: 1) to determine whether the gene signature associated with type 1 cell-mediated cytotoxic immunity was correlated with overall survival, 2) to determine whether WISP1 expression is increased in invasive breast cancer, and 3) to determine whether a pattern of gene expression consistent with inhibition of IL12 signaling axis correlates with WISP1 expression.

## Results

### Type 1 cell-mediated immunity correlates with improved survival in patients with invasive triple negative breast cancer

In this retrospective cohort study, our first objective was to determine whether there were distinct cohorts within the invasive breast cancer arm of the TCGA study that can be defined based upon type 1 cell-mediated immune response. Samples included in the analysis were limited to those derived from patients diagnosed with variants of invasive breast cancer (n = 520) and to those obtained from normal breast tissue (n = 61) (see Dataset S1). As listed in Table 1, normalized expression values were obtained for genes that are associated with T cells, macrophages, and Natural Killer cells and the functional roles that these cells play in cell-mediated immunity. As macrophages and T cells can enhance or inhibit cell-mediated immunity depending on polarization bias, genes associated with alternative polarization states were also included. Specifically, macrophages within the tumor microenvironment are thought to either promote (M1) or inhibit (M2) cytotoxic cell-mediated immunity [28], [29]. Similarly, effector T cells alter their functional role in coordinating adaptive immunity depending on the cytokines secreted by and the transcription factors expressed by different subsets, which include type 1, type 2, type 17, or T regulatory subsets [30], [31]. We also included genes associated with the IL12 cytokine family, as IL12 links innate to adaptive immunity and other members of this cytokine family have competing effects on immune bias (e.g., [32]).

**Table 1:** Genes associated with type 1 cell-mediated immunity.

Functional Annotation	Genes	Refs
CD4/CD8 T cell surface molecules	CD247 CD3D CD3E CD3G ITGAL ITGB2 ICAM1 CD2 CD28 THY1 PTPRC	[74]
T helper cell polarization		
Th1	CD4 IFNG IL10 FASLG EOMES TBX21	[31]
Th2	CD4 IL4 IL5 IL6 IL10 GATA3 PPARG	[31]
Th17	CD4 IL17A IL17F RORA RORC	[31]
iTreg	CD4 TGFB1 IL10 IL12A EBI3 RORC FOXP3 TBX21 CCR6 MYB	[31]
IL12 and Stat4 Dependent Signaling Pathways in Th1 Development	CD247 CD3D CD3E CD3G JAK2 CCR5 CXCR3 ETV5 IFNG IL12RB1 IL12RB2 IL12A IL12B IL18 IL18R1 JUN MAPK14 MAPK8 MAP2K6 STAT4 TYK2	[74]
CTL mediated immune response against target cells	CD247 CD3D CD3E CD3G CD8A FAS FASLG B2M GZMB ITGAL ITGB2 ICAM1 HLA-A PRF1 HLA-B HLA-C	[74]
Natural Killer Cells	KLRC1 KLRC2 KLRC3 KLRD1	–
Natural Killer Cell mediated cytotoxicity	IFNA1 IFNA2 IFNG CD247 FAS FASLG GZMB ICAM1 KLRC1 KLRC2 KLRC3 KLRD1 PRF1 ITGAL ITGB2	[75]
NO2-dependent IL12 pathway in NK cells	CD247 CD2 CD4 JAK2 CCR5 CXCR3 IFNG IL12RB1 IL12RB2 IL12A IL12B NOS2 STAT4 TYK2	[74]
Macrophages	CD14 MRC1 CPM ITGAM NOS2 HLA.DRA HLA.DMA HLA.DOA HLA.DPA1 HLA.DQA1 HLA.DQA2	––
Tumor Associated Macrophages		
M1	IDO1 IL23A IL12B CCL17 IL1B	[29]
M2	ARG1 TIMP2 LYVE1 KLF4 CD163 STAB1	[29]
IL12 Family of Cytokines	IL12A IL12B IL23A EBI3	–
Additional Immunosuppressive Mechanisms	CD274 PDCD1; CTNNB1 WISP1; HMGB1; HIF1A; BTLA; HAVCR2 (TIM-3); LAG3; TGFB1; MICA MICB; VTCN1 (B7-H4)	(see Figure S5)
Cell Proliferation	MKI67	–

**Figure 1:**
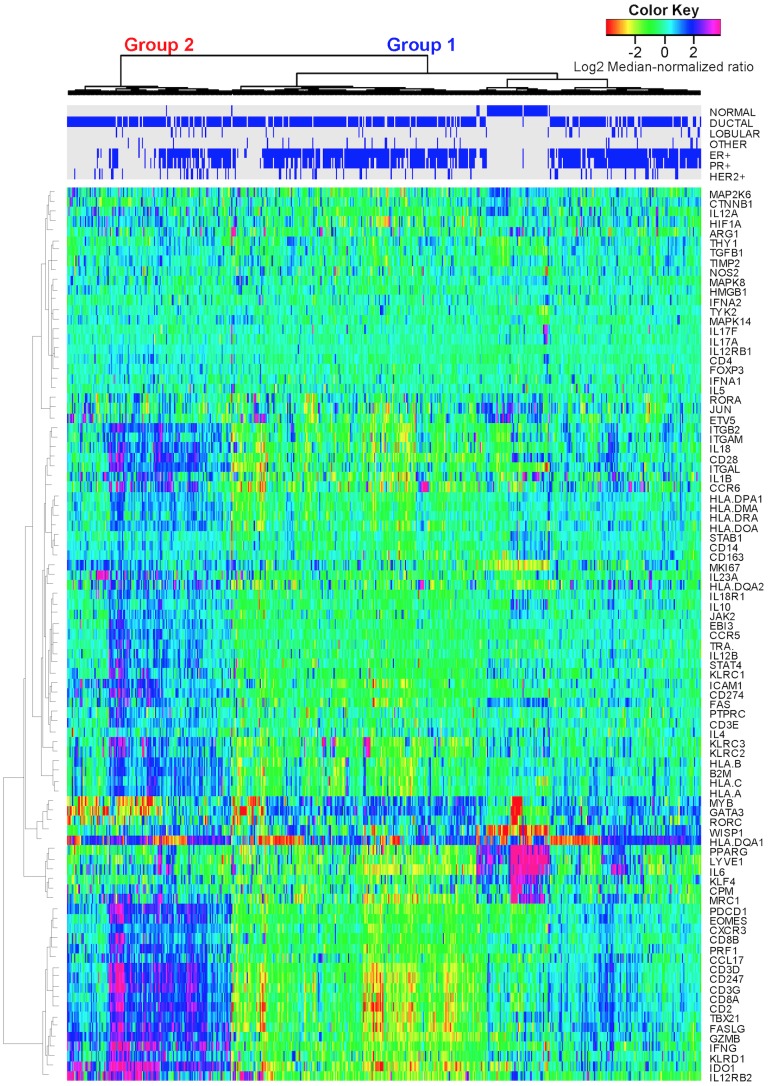
Expression patterns of genes associated with cell-mediated immunity clustered into two main groups. mRNA expression obtained from normal breast and invasive breast carcinoma tissue samples (columns) acquired as part of the Cancer Genome Atlas study [14] were hierarchically clustered into two groups based upon the log2 median-normalized expression ratio for genes (rows) related to cell-mediated cytotoxic immunity and tumor immunosuppression, as listed in Table 1. Tissue samples were characterized by morphology (i.e., normal, ductal, lobular, or other) and molecular histology (i.e., expression of the estrogen receptor (ER+), progesterone receptor (PR+), or HER2 (HER2+)), as highlighted by the blue bars on top. Gene expression is shown as a row-normalized heatmap. Red denotes under-expressed and violet denotes over-expressed relative to the population mean. Dendrogram indicates the degree of similarity among genes (rows) or samples (columns) using the Ward's minimum distance method in R.

**Figure 2:**
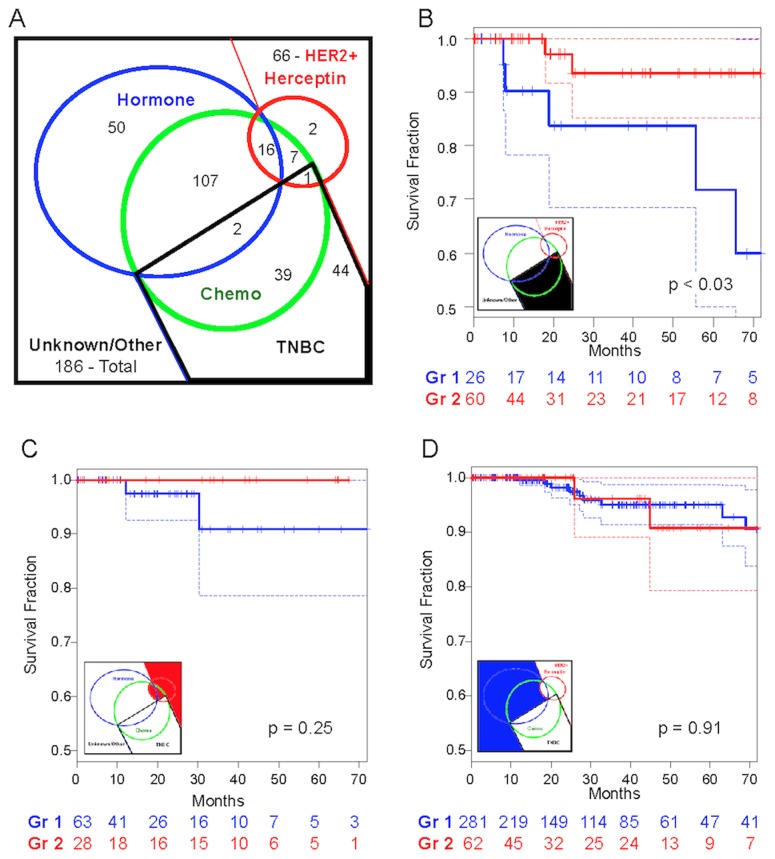
Triple negative breast cancer patients with enhanced type-1 cell-mediated immunity exhibit an improved 6-year survival. Adjuvant treatment of breast cancer is stratified based upon the molecular histology, as summarized by a Venn diagram for this cohort (A). While treatments were documented for 224 of the 520 patients in the cohort, the distribution of known adjuvant therapies are indicated by the color circles (Hormone therapy - blue circle, chemotherapy - green circle, and Herceptin immunotherapy - red circle). Kaplan-Meier survival curves were obtained for invasive breast cancer patients that were stratified by group membership (group 1 - blue curves, group 2 - red curves) within a common adjuvant treatment group. The treatment groups included triple negative breast cancer (B), patients treated with adjuvant Herceptin plus HER2+ patients with unknown treatment (C), and patients treated with adjuvant hormone therapy plus other HER2- patients (D). Dotted lines indicate 95% confidence bounds. The number of patients at risk as a function of time are shown below the x-axis for each Kaplan-Meier curve. A p–value of less than 0.05 was considered statistically significant.

Based upon immune-related gene expression, tissue samples hierarchically clustered into two main groups (Figure 1 and Figure S1). While more patients were associated with Group 1 (n = 370 versus n = 150), the two groups had similar patient population characteristics with no difference between age, tumor stage, menopause status, or lymph node status (Table 2). Based on either molecular pathology or PAM50 intrinsic subtypes [33], the molecular characteristics of tumors associated with these two groups were significantly different (p–value<1x10^-4^ - Table 2). Triple negative (TN) breast cancer samples were significantly enriched in group 2 with an odds ratio of 3.48 versus 0.38 for group 1. As expected, samples positive for either estrogen receptor (ER) or progesterone receptor (PR) and negative for HER2 were enriched in group 1. Given that molecular subtyping guides therapy selection, 6-year overall survival was estimated using Kaplan-Meiers curves for group 1 versus group 2 cohorts stratified by adjuvant treatment, if known, and by molecular pathology (Figure 2). Patients with TN breast cancer that clustered with group 2 exhibited an increase in overall 6-year survival compared with patients with TN breast cancer that clustered with group 1 (p–value<0.03 with hazard ratio = 0.191 (95% C.I. 0.037–0.995)). While the number of events is low for the TN breast cancer group, the overall trend in survival was also observed at earlier time points (1-year survival p–value<0.032 with hazard ratio<0.01; 3-year survival p–value = 0.126 with hazard ratio = 0.28; and 5-year survival p–value = 0.054 with hazard ratio = 0.22). Patients with HER2+ breast cancer also exhibited a similar trend, but the difference in overall survival did not reach a similar level of significance. In contrast, no difference in 6-year survival was observed between the group 1 and group 2 cohorts treated using adjuvant hormone therapy, if known, or were positive for either ER or PR.

**Table 2:** Characteristics of the patient population stratified by group membership.

Category	Group 1	Group 2	Tumor Stage^‡^	Group 1	Group 2	LN Status^‡^	Group 1	Group 2
Count	370	150	O	1	0	N0	89	49
Age (years)^†^	58.2	57.3	I	32	10	N0(i−)	65	32
95% CI	34–82	35–83	IA	28	13	N0(i+)	13	3
			IB	5	1	N1	44	15
Molecular Pathology*			IIA	117	65	N1a	60	18
ER+/PR+	300	75	IIB	83	26	N1b	16	2
HER2+	48	27	IIIA	59	17	N1c	1	1
TN	26	60	IIIB	12	3	N1mi	11	1
Odds Ratio*	0.38	3.48	IIIC	11	8	N2	21	11
95% CI	0.23–0.61	2.26–5.37	IV	11	3	N2a	20	9
			X	11	4	N2b	0	0
PAM50 Intrinsic Subtypes*						N3	9	1
Basal	30	65				N3a	12	6
HER2-like	30	27				N3b	0	0
Luminal A	203	24				N3c	0	1
Luminal B	99	27				NX	9	1
Normal-like	4	4						
**Menopausal status^‡^**								
Pre	78	34						
Peri	14	3						
Post	180	73						
Unknown	98	40						

Statistical significance between group summary statistics was estimated using a two-sided unmatched Student's t test († indicates p–value = 0.48). A Fisher's Exact test was used to test whether categorical data for group is different than population (‡ indicates p–value greater than 0.5, * indicates p–value less than 1x10^-4^). Age summarized as mean and range that encloses 95% of the population. Odds ratio calculated based upon diagnosis of TN breast cancer within each cohort group.

LN = Lymph node.

**Figure 3:**
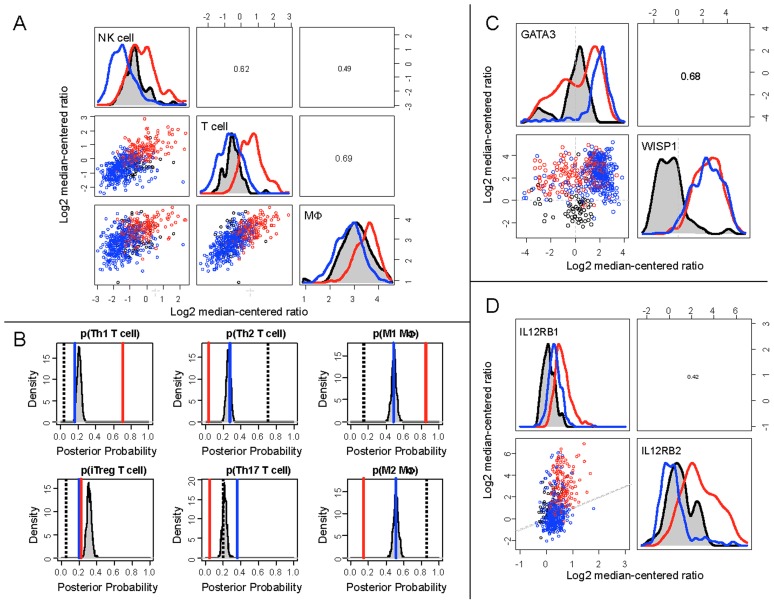
Gene expression clusters associated with invasive breast cancer exhibited differential cell-mediated immune response relative to normal breast tissue. Relative immune cell infiltrate was estimated based upon the average expression of genes associated with NK cells, T cells, and macrophages, as listed in Table 1 (A). Mean posterior probability associated with T helper cell and macrophage polarization in each group (Group 1 - blue line, Group 2 - red line, Normal - black dotted line, random gene expression (null hypothesis) - gray shaded density distribution) were estimated based upon mutually exclusive gene expression patterns that are associated with each cell subset, as also listed in Table 1 (B). WISP1 expression was increased in breast cancer relative to normal tissue samples and WISP1 expression correlated with GATA3 expression (C). The ratio of IL12RB2 to IL12RB1 was increased in Group 2 patients relative to Group 1 patients and normal breast tissue samples (D). A 1∶1 ratio between IL12RB2 and IL12RB1 gene expression is indicated by the gray dotted line in the scatter plot. In panels A, C, and D, bivariate scatter plots are shown below the diagonal, marginalized histograms stratified by the three groups are shown on the diagonal, and correlation coefficients are shown above the diagonal. Results are colored by group (Breast Cancer Group 1: blue, Breast Cancer Group 2: red, Normal breast tissue: black).

Effective anti-tumor immunity depends on the product of the number of immune cells present within the tumor microenvironment and the efficacy of the immune cells present to elicit cell-mediated cytotoxic immunity. To infer mechanistic differences in anti-tumor immunity between the patient cohorts, we estimated the magnitude and the quality of anti-tumor immunity from the gene expression measurements. The relative magnitude of immune cell infiltration was inferred from the average expression values for genes associated with NK cells, T cells and macrophages (Table 1). Compared to samples derived from normal tissues, the group 1 cohort exhibited a gene expression signature associated with reduced NK cell, T cell, and macrophage recruitment (Figure 3A). In contrast to group 1, the immune cell signature in the group 2 cohort suggested an increase in NK cell, T cell, and macrophage recruitment relative to normal breast tissue. As immune cell polarization influences the efficacy of anti-tumor immunity, we used model-based inference to determine the polarization signature. In normal breast tissue, T helper cells were primarily polarized towards Th2 (p–value<0.001) and macrophages were polarized towards a M2 phenotype (Figure 3B, p–value<0.001). In invasive breast cancer, group 1 cohort exhibited a mixed Th2 and Th17 immune bias and a strong Th1 bias was associated with group 2 samples (p–value<0.001). Consistent with the Th1 bias in group 2 samples, the genes associated with a type 1 cell-mediated immune response were also consistently expressed at higher levels in the group 2 cohort compared to group 1 and normal tissue samples. Compared to the null hypothesis for immune cell polarization, the macrophage polarization bias in the group 1 cohort could not be distinguished from a pattern of random gene expression and samples from the group 2 cohort exhibited a strong M1 bias (p–value<0.001). Collectively, the genes associated with type 1 cell-mediated immunity can be used to identify cohorts within invasive breast cancer that correlate with improved 6-year survival, specifically in patients with TN invasive breast cancer.

### Increased WISP1 associates with oncogenic transformation in invasive breast cancer

**Figure 4:**
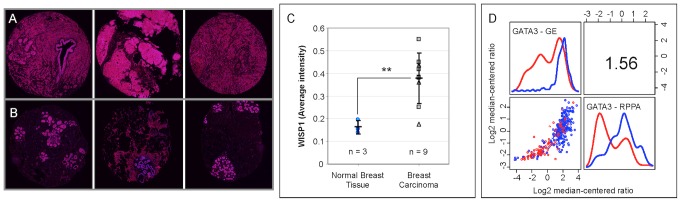
WISP1 and GATA3 gene expression correlate with protein expression. Representative deconvoluted color images derived from a breast cancer tissue microarray probed using a WISP1 antibody and imaged using 3,3′ diaminobenzidine and stained using hematoxylin for three invasive breast cancer (A - top) and three normal breast (B - bottom) tissue samples (original tissue microarray images were obtained from www.proteinatlas.org [64]). Deconvoluted intensity of WISP1 staining is shown in red while cellular structures stained using hematoxylin are shown in blue. (C) The average intensity of WISP1 staining, as determined by color deconvolution of the RGB tissue microarray images, was increased in breast carcinoma (** indicates a p–value<0.001, as estimated using an unpaired two-tailed Student's t-test). Summary statistics are shown as mean +/− SD. The number of replicates included in the analysis is indicated by n. (D) GATA3 gene expression as quantified by Affymetrix microarray correlated with GATA3 protein expression as quantified by reverse phase protein array. In panel D, a bivariate scatter plot for the 340 joint measurements is shown below the diagonal, marginalized histograms stratified by the two breast cancer groups are shown on the diagonal, and a correlation coefficient is shown above the diagonal.

Principal component analysis (PCA) was used to gain insight into the molecular differences between cohorts identified using hierarchical clustering. As the samples from normal tissues clustered predominantly with group 1, the group 1 cohort was subdivided into three subsets (Figure S2A) with normal tissue samples clustering into group 1a. Of the total variation contained within the gene expression data, the first four principal components (PCs) captured 54% of the variance (Figure S2B). Differences among the clustered cohorts were observed for the first four PCs while no significant differences among groups were observed for the rest of the PCs (Figure S2C). Based upon the loading coefficients for individual genes, the magnitude of PC1 corresponded to the extent of T cell-mediated (increases with *CD2*, *CD247*, *CD3G*, *CD3D*, *CD8A*, and *CD28* expression) type 1 cytotoxic immunity (increases with *FASLG, IFNG, GZMB, TBX21, IL12RB2, EOMES, PRF1, B2M* and decreases with *GATA3* expression) and PC2 captured a correlation between *WISP1* and the T cell lineage-defining transcription factors *GATA3* and *PPARG*. As described in the supplemental Text S1, a similar gene expression signature was observed in the gene expression results reported by Gluck et al. (GSE22358 [34]). PC projections of the patient samples suggest that the extent of T cell-mediated cytotoxic immunity within invasive breast cancer is a continuous property, with TN breast cancer more prevalent at higher values for PC1 and HER2+ breast cancer more prevalent at lower values for PC1 (Figure S2C). Yet, the hierarchical clustering subdivided this continuous property into two discrete cohorts. In contrast to PC1, PC2 separated samples derived from invasive breast cancer from normal breast tissue and, based upon the loading coefficients for PC2, suggests that an increase in *WISP1* expression correlates with oncogenic transformation (Figure 3C - p–value<1x10^-15^). The average intensity of WISP1 antibody staining in an independent tissue microarray that contained samples from normal (n = 3) and breast carcinoma tissue (n = 9) were used to validate that an increase in WISP1 correlates with oncogenic transformation (Figure 4, panels A–C). The tissue microarrays were consistent with the gene expression data such that WISP1 was increased in invasive breast cancer compared to normal breast tissue (p–value<0.001).

### An increase in WISP1 correlates with a shift in immune bias from Type 1 towards Type 2 immunity

T cell polarization is driven by competition among lineage-defining transcription factors that are induced by the exogenous action of polarizing cytokines [30]. By inducing T-bet, Interleukin-12 (IL12) acts, in part, upon CD4^+^ T helper cells to organize an effective type 1 cell-mediated immune response [18]. As a potential inhibitor of a type 1 cell-mediated immune response, in vitro co-culture assays identified WISP1 as a paracrine regulator of immune cells by inhibiting response to IL12 [27]. Here, we found that the loading coefficients associated with PC2 suggest that the variation in *WISP1* was also associated with two T cell lineage-defining transcription factors: *GATA3* and *PPARG*. Specifically, *WISP1* expression was correlated with *GATA3* expression (Figure 3C - p–value<1x10^-9^) and exhibited a negative correlation with *PPARG* expression (see Figure S4A - p–value<1x10^-15^). The functional connection between WISP1 and immune polarization is strengthened by the observations that *GATA3* expression also correlates with GATA3 protein abundance (Figure 4D - p–value<1x10^-15^) and that *GATA3* expression does not correlate with changes in genome copy numbers of *GATA3* (Figure S4B - p–value=1). In addition, *GATA3* expression exhibited a negative correlation with IL12 receptor *β* 2 (Figure S4C - p–value<1x10^-15^). Compared to other putative immunosuppressive mechanisms present in the tumor microenvironment, the WISP1-GATA3 signature was the only mechanism that was higher in the group 1 cohort relative to the group 2 cohort (see Figure S5).

Peroxisome proliferator-activated receptor 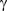 (PPARG) is a ligand-activated transcription factor that plays an important role in regulating immunity and oncogenesis [35]. For instance, Th2 polarization is associated, in part, with increases in *GATA3*, *IL6*, and *PPARG* gene expression [31]. In contrast, Chung et al. report that PPARG can form an inhibitory complex with nuclear factor of activated T cells (NF-AT) that inhibits the transcription of IL4 in T helper cells [36]. In a mouse model of atopic asthma, pharmacologic activation of PPARG reduces the canonical Th2 cytokines IL4 and IL13 and GATA3 protein in lung extracts [37]. Collectively, these results suggest that the gene set - *CD4*, *IL4*, *IL5*, *IL10*, and *GATA3* - is a better signature for a type 2 bias of T helper cells as an increase in *PPARG* expression may sensitize Th2 effector cells to negative regulation by PPARG ligands and that *GATA3* and *PPARG* may be regulated independently. Using this revised Th2 cell gene signature, the group 1 cohort is associated with an increase in type 2 bias relative to the group 2 cohort and normal breast tissue (Figure S6 - p–value<0.001). The group 2 cohort exhibited a strong type 1 bias while a Th17 bias was observed in samples from normal breast tissue. An expression signature consistent with inducible T regulatory cells was not observed in any of the cohorts (p–value<0.001). Using a Cox proportional hazards regression model, we also found that polarization towards a T helper type 1 phenotype was a predictor of survival independent of the molecular pathology (see ). Using bootstrap resampling of the genes listed in Table 1, the model-based inference of type 1 polarization is a better predictor of improved survival than 95% (i.e., p–value<0.05) of the random immune signatures for the 1 Yr, 3 Yr, and 6 Yr outcomes and than 93% of the random signatures for 5 Yr outcome (see Figure S7).

### Alterations in IL12 receptor expression and MHC class I abundance between groups are retained in representative breast cancer cell lines

**Figure 5:**
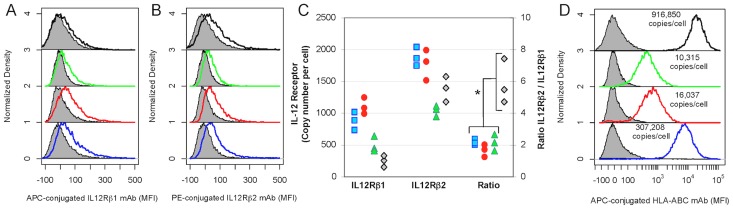
Basal copy numbers of IL12RB1, IL12RB2, and MHC class I in breast cancer cell lines were consistent with cohort group assignment based upon molecular pathology. Copy numbers of membrane proteins IL12RB1 (A), IL12RB2 (B) and HLA-ABC (D) were quantified using flow cytometry for four breast cell lines: 184A1 HMECs (blue), BT474 (HER2+/ER+/PR+) cells (red), SKBR3 (HER2+/ER−/PR−) cells (green), and MDA-MD-231 (HER2−/ER−/PR−) cells (black). Unstained cells are shaded in gray. Results are representative of three independent replicates. (C) The ratio of IL12RB2/IL12RB1 was increased in MDA-MB-231 cells relative to the other three cell lines (* indicates a p–value<0.05, as estimated using an unpaired two-tailed Student's t-test).

In addition to secreting WISP1, B16 model for melanoma also overexpresses one component of the IL12 receptor, IL12R*β*2, that, in vitro, creates a local cytokine sink for IL12 [27]. We have also found that STAT4 is phosphorylated irreversibly, creating a short term memory to IL12 signaling that is limited by cell proliferation [38]. Local delivery of IL12 to the tumor microenvironment promotes tumor regression in the B16 melanoma model [24] and in the EL4 thymoma model [25]. Collectively, these studies imply that signaling by endogenous IL12 within the tumor microenvironment helps to maintain T cell polarization when cognate tumor antigens induce T cell proliferation [39] and that manipulating this extracellular control mechanism may impart a survival advantage to the collective tumor population. Finally, we wanted to determine whether IL12 receptor *β*2 was increased relative to IL12 receptor *β*1 in samples derived from tumors with active type 1 cell-mediated immune response. In invasive breast cancer, the ratio of *IL12RB2* to *IL12RB1* expression was increased in the group 2 cohort relative to the group 1 cohort (Figure 3D - p–value<1x10^-15^). As we had previously observed an imbalance in copy numbers of the components of the IL12 receptor in malignant melanocytes, we measured IL12 receptor *β*1 and IL12 receptor *β*2 copy numbers in the 184A1, BT474, SKBR3, and MDA-MB-231 cell lines by flow cytometry (Figure 5, panels A–B). While the copy numbers in these cell lines were lower than what we had observed in the B16F0 and B16F10 cell lines, the ratio of IL12 receptor *β*2 to IL12 receptor *β*1 was increased in the triple-negative breast cancer model, MDA-MB-231, relative to the other three cell lines (see Figure 5C - p–value<0.05).

We also observed that abundance of major histocompatibility complex (MHC) class I molecules varied among these cell lines in a pattern consistent with differences in type 1 cell-mediated immunity (Figure 5D). Specificity in directing T cell-mediated cytotoxic immunity depends on the presentation of peptides bound to MHC class I molecules. A reduction in MHC class I expression may reduce the efficacy of type 1 cell-mediated immunity in controlling tumor growth. Given that the gene expression signature associated with type 1 cell-mediated immunity varied among the invasive breast cancer samples, we ascertained whether there were any basal differences in copy numbers of MHC class I among cell lines derived from the different cohorts. The BT474 and SKBR3 cell lines are cell models of HER2+ breast cancer, a subset that exhibited a gene expression signature associated with a low type 1 cell-mediated response. The 184A1 cell line is a cell model of normal breast tissue, which had an intermediate type 1 cell-mediated response signature. The MDA-MB-231 cell line is a cell model of triple negative breast cancer, which exhibited a high type 1 cell-mediated response signature. Copy numbers of MHC class I molecules were assayed by flow cytometry (Figure 5D). Collectively, the copy numbers of MHC class I molecules varied among the cell lines: 184A1 expressed 307K copies while BT474 and SKBR3 expressed lower copies (16K and 10K, respectively) and MDA-MB-231 expressed higher copies (916K). The lower copies in the BT474 and SKBR3 cell lines is consistent with previous studies that report that over-expression of HER2/Neu reduces MHC class I expression [40], [41]. In summary, basal differences in MHC class I and IL12 receptor copy numbers among the 184A1, BT474, SKBR3, and MDA-MB-231 cells lines are consistent with a model of invasive breast cancer where the molecular subtypes are distinguished by differences in type 1 cell-mediated immunity.

### Existing genetically engineered mouse models for spontaneous breast cancer do not reproduce immune cell gene signature observed in TCGA data

Modeling breast cancer in mice inevitably involves some degree of abstraction - one must determine key elements associated with the human disease and select model systems that incorporate those elements. Historically, transplantable models for cancer, like the B16 melanoma and 4T1 breast cancer models, have been used to study anti-tumor immunity *in vivo*. Cell lines that were derived from spontaneous tumors can be manipulated *in vitro* to express defined tumor antigens and re-introduced into a syngeneic host. However, transplantable models have been criticized as they do not resemble established spontaneous tumors (e.g., [42], [43]). One of the advances associated with pre-clinical drug discovery and development has been the development of genetically engineered mouse models (GEMM) for cancer that incorporate alterations in known oncogenes and tumor suppressors. Breast cancer GEMMs spontaneously develop lesions in mammary tissue that histologically resemble the human equivalent [44]. Yet GEMMs are not without criticism, as Jacks and coworkers suggest that genetically engineered mouse models of cancer may underestimate the mutational and antigenic load of most human cancers [45]. Given the observed immune gene expression signature observed in human breast cancer tissue, we also wanted to determine whether most common breast cancer GEMMs exhibit similar immune gene signatures as the human disease.

**Figure 6:**
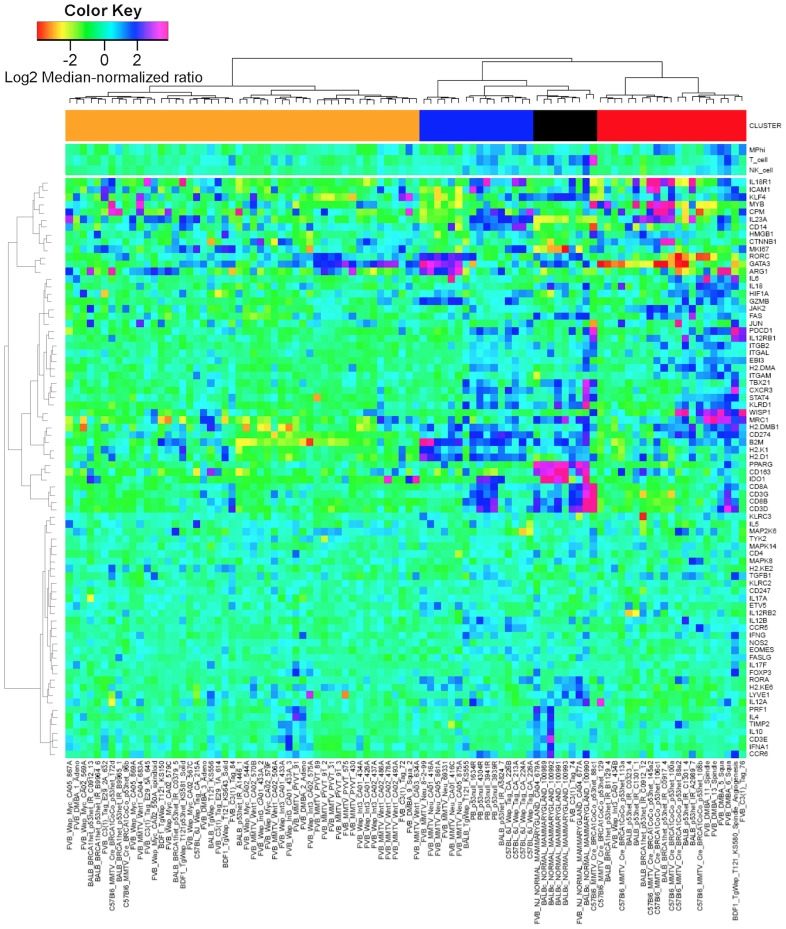
Immune gene expression signatures in genetically engineered mouse models for breast cancer clusters into three groups. mRNA expression obtained from normal and breast cancer carcinoma tissue samples derived from different genetically engineered mouse models (columns) were hierarchically clustered into four groups based upon the log2 median normalized expression ratio for genes (rows) related to cell-mediated cytotoxic immunity and tumor immunosuppression. The color-coded bar at the top of the heatmap indicates the four groups (Normals - black, Group 1 - blue, Group 2 - red, and Group 3 - orange). Gene expression is shown as a row-normalized heatmap, where red denotes under-expressed and violet denotes overexpressed relative to the population mean. Dendrogram indicates the degree of similarity among genes (rows) or samples (columns) using the Ward's minimum distance method in R.

**Figure 7:**
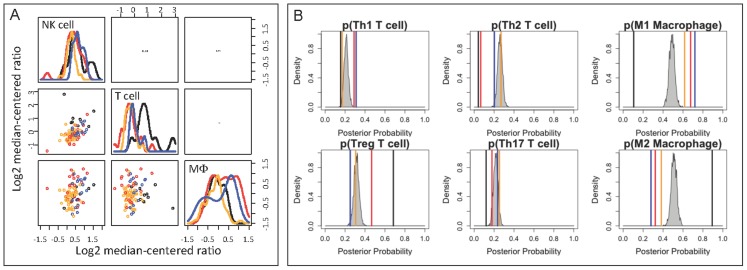
Hierarchical clusters of GEMM gene expression exhibit different immune cell signatures. (A) Relative immune cell infiltrate was estimated based upon the average expression of genes associated with NK cells (KLRD1, KLRC2, KLRC3), T cells (CD247, CD3G, CD3D, CD3E), and macrophages (CD14, CPM, MRC1, ITGAM) in the breast cancer GEMM data set [46]. (B) Using model-based inference, the mean posterior probabilities associated with T helper cell and macrophage polarization in each GEMM group were estimated based upon mutually exclusive gene expression patterns that are associated with each cell polarization subset, as listed in Table 1. The immune polarization signature was compared to a signature assembled from a bootstrapped ensemble of random sets of gene expression (i.e., a null hypothesis), as summarized by the gray shaded density distribution. In both panels, results are colored by group indicated at the top of the GEMM heatmap shown in Figure 6 (Normals - black, Group 1 - red, Group 2 - blue, and Group 3 - orange). In panel A, bivariate scatter plots are shown below the diagonal, marginalized histograms stratified by the three groups are shown on the diagonal, and correlation coefficients are shown above the diagonal.

Similar to the TCGA analysis presented in Figure 1, mRNA expression results from 122 breast cancer tumor and normal breast tissue samples obtained from a variety of genetically engineered mouse models (GEMM) for breast cancer (see Figure 6 - [GEO:GSE3165]) [46]. Within this GEMM data set, four gene expression clusters were identified based upon a subset of genes associated with anti-tumor immunity and immunosuppressive mechanisms: a normal group and three breast cancer groups. In contrast to the TCGA data, *WISP1* was up-regulated in only a small subset of 7,12-dimethylbenz[a]anthrazene (DMBA)-induced breast cancer models. Moreover, the immune gene expression signatures in the breast cancer GEMMs suggested that NK cell infiltrate is unchanged, T cell infiltrate is suppressed, and that macrophages are elevated relative to normal breast tissue (see Figure 7A). This signature is different from the immune cell gene signatures observed in the TCGA data set that suggest that NK cells, T cells, and macrophages were either collective decreased in group 1 or collectively increased in group 2 relative to normal tissues. In terms of immune polarization, macrophages exhibit a similar pattern in the GEMMs compared to the human samples, where macrophages in normal tissue are skewed towards an M2 and macrophages in tumor tissue are skewed towards M1 phenotypes (see Figure 7B). In contrast, the inferred T cell polarization states in GEMMs are different from human samples. A regulatory T cell signature is highest in normal mammary tissue and the different tumor models exhibit mixed immune polarization signatures that overlap with the null hypothesis signature. The GEMM results also suggest that reduced T cell recruitment to mammary tumors in the GEMMs reduces the signal associated with T cell polarization, which in turn makes identifying the T cell polarization state from homogenized tissue samples difficult.

Similar discordance has been reported between human inflammatory diseases and their corresponding mouse models [47]. Collectively, the immune signatures in the set of GEMMs are unlike that observed in the TCGA samples and motivate developing pre-clinical mouse models that better reflect the immune signature associated with invasive breast cancer in humans. Given the discordance between GEMMs and humans, the likelihood for a type 2 error in testing immunotherapies using spontaneous mouse models for breast cancer seems high. Unfortunately, developing GEMMs that better mimic human anti-tumor immunity is a recommendation that has persisted for over three decades [48]. In contrast to the 1980s, this work illustrates how data obtained from large-scale studies, like the Cancer Genome Atlas, coupled with *in silico* model-based inference methods can be used to identify appropriate immune signatures. These immune signatures can be used as design constraints in genetically engineering better pre-clinical mouse models of cancer.

## Discussion

In this retrospective study of the invasive breast cancer arm of the Cancer Genome Atlas study, we made three main observations. First, type 1 cell-mediated cytotoxic immunity was correlated with increased survival in patients with invasive TN breast cancer and was predictor of survival independent of molecular pathology. In the absence of a molecular targeted therapy, an increase NK and T cell infiltrate and a shift from type 2 towards type 1 cell-mediated immunity correlated with a survival advantage in patients with invasive TN breast cancer. These correlates were also identified independent of tumor staging and lymph node involvement. This observation is consistent with a significant body of literature that correlate various molecular characteristics of anti-tumor immunity with overall survival in breast cancer, as reviewed by Andre et al. [49]. While many studies focus on particular immune-related molecules, gene expression profiling provides a less biased view of the immunologic properties of anti-tumor immunity. As one of the most extensively studied cancers, gene expression profiling of primary tumors (e.g., [50]–[54]) and tumor stroma [55] have identified immune classifiers of clinical outcome in breast cancer. In contrast to a focus on primary tumors, samples from age-matched normal breast tissue were included in the analysis to gain insight into the immunologic changes associated with oncogenic transformation, which led to the second observation.

Second, oncogenic transformation in invasive breast cancer was associated with an increase in WISP1 gene expression (p–value<1x10^-15^) and protein abundance (p–value<0.001). Prior studies of WISP1 in breast cancer have been mixed. Xie and coworkers observed higher WISP1 gene expression in 20 of 44 breast cancer samples while the remaining samples exhibited levels of expression similar to normal breast tissue [56]. In contrast, Davies and coworkers found that WISP1 was reduced in samples obtained from breast tumors compared to normal breast tissue [57]. Here, the focus on invasive breast cancer and larger sample size may explain some of the observed differences. WISP1 is a member of the family of connective tissue growth factors that is induced by nuclear localization of beta-catenin [26] and participates in stem cell differentiation and tumorigenesis [58]. While the details remain to be fully elucidated, on-going work suggests that proteolytic cleavage of E-cadherin enables membrane-bound beta-catenin to localize to the nucleus and induce WISP1 expression [27]. In vivo, loss of E-cadherin plays an important role in the metastatic potential of cancers [59]. While WISP1 has been reported to influence neurodegeneration and osteogenesis [60], a signaling mechanism has yet to be identified despite a report suggesting that WISP1 binds the 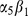 integrin [61].

Third, the gene expression signature in invasive breast cancer was consistent with WISP1 as a paracrine inhibitor of type 1 cell-mediated immunity through inhibiting IL12 signaling and promoting type 2 immunity. In particular, we found a highly significant correlation between *WISP1* and *GATA3* (p–value<1x10^-9^) and a highly significant negative correlation between *WISP1* and *PPARG* (p–value<1x10^-15^). While the increase in GATA3 is consistent with WISP1 as an inhibitor of IL12 signaling, the negative correlation between WISP1 and PPARG was unexpected and suggests that the reduction in PPARG may relieve transcriptional repression of type 2 cytokine production by T cells. Conventionally, exploratory data analysis is used to identify genes or clusters of genes that correlate with clinical outcome (e.g., [50]–[55]). To identify gene set classifiers, the gene expression data set is subdivided into a training set - to discover significant gene clusters - and a validation set - to confirm that the gene cluster correlates with outcome. Here a more targeted approach was used. Previously, we used a phenotypic assay that incorporated *in vitro*, *in silico*, and unbiased proteomics methods to discover a paracrine mechanism by which a mouse model for melanoma regulates immune response to IL12 [27]. To validate this mechanism, a retrospective analysis was used to identify whether a gene expression signature that is consistent with WISP1 as a paracrine regulator of anti-tumor immunity exists in invasive human breast cancer. This gene signature was embodied in PC2 and corresponded to oncogenic transformation. Inverse relationships between type 1 cell-mediated cytotoxic immunity and *GATA3* and between *IL12RB2* and *GATA3* were also captured in PC1 and PC3, respectively. Collectively, the first three PCs captured 49% of the overall variance in gene expression.

The limitations of the analysis are such that the TCGA data reflect homogenized tumor tissue and the genes that are associated with the immune polarization signatures have pleiotropic biological roles. For instance, GATA3 plays a role in both mammary epithelial [62] and immune cell biology [63]. In terms of mammary epithelial cell biology, GATA3 is thought to inhibit breast cancer metastasis. GATA3 is up-regulated in luminal epithelial cells and down-regulated in basal epithelial cells, which have a higher propensity for invasion and metastasis. In this TCGA study, mutations in *GATA3* have a higher prevalence [14]. However, less than 2% of samples that exhibited basal-like and HER2-enriched intrinsic subtype characteristics had mutations in *GATA3*, specifically truncation mutations. TN breast cancer samples were predominantly the basal-like subtype while HER2+ samples corresponded to the HER2-enriched subtype. In terms of immune cell biology, GATA3 promotes type 2 and counter regulates type 1 T cell polarization, as captured in the analysis by the negative loading coefficient for *GATA3* in PC1 and the reciprocal relationship between *GATA3* and *IL12RB2* in PC3. Collectively, the data suggest that changes in *GATA3* may reflect a signature derived from immune cell biology rather than mammary epithelial cell biology. We found that *GATA3* expression was correlated with *WISP1* and that *GATA3* was up-regulated in invasive breast cancer compared to normal tissue. The increase over normal in *GATA3* was especially prevalent in patient samples associated with the group 1 cohort, a cohort with reduced overall survival for patients with TN breast cancer. In contrast, *GATA3* expression was lower relative to normal tissue in a subset of the group 2 cohort, a cohort with improved overall survival for patients with TN breast cancer. Despite the increase in WISP1 associated with oncogenesis, the differences in immune bias between the patient cohorts may reflect intrinsic differences in sensitivity to local reprogramming of tumor-infiltrating lymphocytes. While this cross-sectional tumor biopsy study can not rule out the possibility that the observed signatures are due to systemic alterations in immune response, identifying local factors could help improve the efficacy of many of the immunotherapies currently in clinical trials, such as adoptive T cell transfer or immune checkpoint inhibitors that increase systemic T cell numbers.

In summary, effective anti-tumor immunity is proportional to the number and to the cytotoxic activity of immune cells that enter the tumor microenvironment. Recent advances in cancer immunotherapy stem from increasing the number of tumor-infiltrating immune cells by inhibiting immune checkpoints or adoptive T cell therapy. Mirroring the clinical results of these therapies, we found that a gene signature consistent with enhanced type 1 cell-mediated cytotoxic immunity is a predictor of overall survival in invasive breast cancer independent of molecular pathology. In addition, this study also supports a link between epithelial-to-mesenchymal transition - through secretion of WISP1 - and repression of type 1 cell-mediated cytotoxic immunity - through inhibition of IL12 signaling. From an evolutionary perspective, the results also suggest that oncogenic transformation in invasive breast cancer alters the selective fitness landscape through reducing the efficacy of innate and adaptive immunity, which function as important extracellular control mechanisms. Restoring these extracellular control mechanisms will require a better understanding of the dynamics associated with the shift in polarization from type 1 to type 2 within tumor-infiltrating lymphocytes and the sensitivity of this axis, in terms of both quantity and quality, to pharmacological action. From a translational science perspective, these findings motivate a directed effort to demonstrate - **using pre-clinical mouse models of invasive breast cancer that more accurately represent the immune signature in human disease** - that inhibiting these paracrine immunosuppressive cues will improve the overall response of current cancer immunotherapies. Moreover, model-based inference helped identify the immune signatures that can be used as design constraints in genetically engineering better pre-clinical mouse models of cancer. This knowledge may be of particular importance to patients with TN breast cancer, a patient group that is underserved by the current generation of molecular targeted therapies.

## Materials and Methods

### Breast tissue gene expression

Expression of genes associated with type 1 cell-mediated immunity, as summarized in Table 1, in normal breast tissue and invasive breast cancer were obtained from the breast cancer arm of The Cancer Gene Atlas (TCGA) study [14]. In brief, homogenized samples obtained from primary tumor (n = 520) and matched normal breast tissue (n = 61) were obtained from patients that were newly diagnosed with invasive breast adenocarcinoma following surgical resection and that received no prior treatment for their disease (chemotherapy or radiotherapy). Subsequent treatments, clinical characteristics and biomarkers, and overall outcome for all of the 520 tumor tissue samples and reverse-phase protein array (RPPA) data for only 340 of the tumor tissue samples were downloaded from the TCGA website (https://tcga-data.nci.nih.gov/tcga/). The median age at diagnosis was 59 years of age and the median follow-up time for overall survival was 22 months. Tumor and normal breast tissue gene expression for this cohort was obtained following array normalization by processing through the Oncomine database (www.oncomine.org). In brief, Level 2 data obtained using Agilent mRNA expression microarrays were downloaded from the TCGA website (https://tcga-data.nci.nih.gov/tcga/) and the intensity of a given gene probe was normalized to the median of the probe intensities across the entire array sample. Expression of a given gene is expressed in terms of a log2 median-centered ratio, where genes that have a value less than zero are expressed at a level less than the median and genes with a value greater than zero are expressed at levels higher than the median.

### Immunohistochemical analysis

The abundance of WISP1 in invasive breast cancer and normal breast tissue was quantified by immunohistochemical analysis using a tissue microarray derived from de-identified human breast tissue samples, as provided by the Human Protein Atlas (www.proteinatlas.org, Stockholm, Sweden [64]) and in accordance with approval from the Uppsala University Hospital Ethics Committee. The tissue microarray analysis included samples from 9 breast adenocarcinomas (6 ductal and 3 lobular) and 3 normal breast tissues from women that ranged in age from 23 to 87 years. The tissue microarrays were processed as described by Uhlen et al. [65] and probed using a rabbit polyclonal antibody against WISP1 (ab10737 - Abcam, Cambridge, MA) that was validated by providing partly consistent staining patterns with another antibody and gene/protein characterization data. WISP1 staining was visualized using diaminogenzidine and microscopic tissue features were visualized by counterstaining with Harris hematoxylin. Immunohistochemically stained tissue microarrays were scanned at 20× resolution (1 mm diameter) and provided as an 8-bit RGB JPEG image. Pathological assessments of the images were annotated manually. The average intensity of WISP1 staining per tissue sample was quantified by deconvoluting the intensity of WISP1 staining from nonspecific hematoxylin tissue staining in R using the *EBImage* package and deconvolution approach described by Ruifrok and Johnston [66].

### Model-based inference and statistical analysis

Polarization of T helper cells and macrophages into different subtypes are defined by differences in gene expression [29], [31]. The genes associated with each subset are summarized in Table 1. The log2 median-centered ratios of subset-defining genes were normalized to the standard deviation of the observed values across the entire cohort, that is a z-score. Immune cell polarization among alternative subsets is assumed to be a mutually exclusive process. Using Bayes theorem, the conditional probability that cells contained within a homogenized tissue sample exhibit a polarization bias, as represented by a model (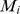), given the observed multi-gene expression signature, 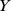, can be express as:
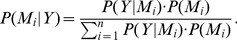
(1)where 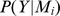 is the likelihood of observing data 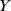 given the polarization model 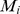, 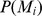 is the prior for the model, and 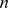 is the number of polarization subsets. As we have equal ignorance *a priori* as to how well the competing polarization models describe the data, the priors for each model are set equal to 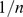. The likelihood of observing increased expression of a defined multi-gene signature (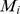) is the product of the likelihood of observing increased expression of each gene (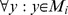) within the signature. The likelihood can be defined using a simple Euclidian metric based upon the z-score [67], [68]:
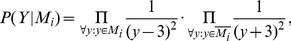
(2)such that a higher z-score for genes (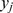) associated with a subset (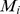) and a lower z-score for genes associated with a different subset (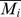) corresponds to a higher likelihood. Bootstrap resampling is an effective computational method for estimating the uncertainty associated with a calculated value [69]. Bootstrap resampling with replacement (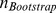 = 1000) from the set of all observed gene expression values was used to establish a predicted polarization bias for an equivalent size patient cohort (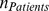 = 200) that is consistent with a null hypothesis, that is the gene expression values are random samples and contain no information regarding immune cell polarization. The distribution in posterior probability of immune bias for a given cohort was obtained using kernel density estimation. Statistical significance associated with the mean posterior probability of immune bias for a given cohort was compared to the null hypothesis. A p–value corresponds to the likelihood that the observed posterior probability (or a more extreme value) of immune bias for a cohort is due to random chance. A p–value<0.05 was considered significant.

The variation and correlation among the gene expression measurements were characterized using principal component analysis (PCA), which is described in more detail in the Supplemental Text S1. The scoring coefficients for each of the top 10 principal components are listed in Dataset S1. Statistical differences between means were assessed using unpaired Student's t-tests. All Student's t-tests were two-sided. Statistical significance associated with a correlation between gene expression values within a sample was assessed using a Pearson product-moment correlation coefficient. A one-sided test of the Pearson's correlation coefficient was used to determine whether a correlation coefficient was positive or negative. A p–value<0.05 was considered significant. Overall survival time was used as a clinical outcome metric. To estimate cumulative survival probability, Kaplan-Meier survival curves were estimated from the cohort overall survival data. Statistical significance associated with a difference in survival between two groups was estimated using the Peto & Peto modification of the Gehan-Wilcoxon test and the Cox proportional hazards regression model, as implemented in the R *survival* package. Based upon criticism of other gene signatures associated with cancer survival [70], the significance of the hazard ratio associated with a type 1 immune polarization bias was estimated by comparing the hazard ratio predicted by type 1 immune polarization model against a distribution in hazard ratios predicted from an ensemble of random models that were created by bootstrap resampling (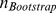 = 1000). Each random model was created by randomly assigning a small subset of genes selected from the genes shown in Table 1 to one of four polarization states. The number of genes associated with each of the four polarization states was the same as listed in Table 1. A p–value corresponds to the likelihood that the observed hazard ratio (or a more extreme value) associated with type 1 immune polarization is due to random chance, where a p–value<0.05 was considered significant. All analyses were performed using R software version 2.14.1 (http://www.r-project.org). Overall, the study was performed in concordance with the REMARK guidelines [71].

### Cell lines, antibodies, and reagents

The nontumorigenic human breast epithelial cell line 184A1 was obtained from ATCC (Manassas, VA), the human HER2+ breast cancer cell lines (BT474 and SKBR3) were kindly provided by Dr. Jia Luo (University of Kentucky; Lexington, KY), and a cell model of triple negative breast cancer (MDA-MB-231) was a gift from Dr. J. M. Ruppert (West Virginia University). The 184A1, BT474, and SKBR3 cell cultures were maintained at 37
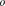
C in 5% CO_2_ in media supplemented as described previously [72]. Similarly, the MDA-MB-231 cell line was maintained in Dulbecco's Modification of Eagle's Medium (DMEM) supplemented with 10% (v/v) heat inactivated fetal bovine serum (FBS) (Hyclone, Inc., Logan, UT), L-glutamine, and penicillin/streptomycin (BioWhittaker, Walkersville, MD). Allophycocyanin (APC)-conjugated mouse anti-human CD212 (IL12R*β*1 - Clone 2.4E6) and APC-conjugated mouse anti-human HLA-A,B,C (Clone G46-2.6) were purchased from BD Biosciences (San Diego, CA, U.S.A.). Phycoerythrin (PE)-conjugated mouse anti-human IL-12 receptor *β*2 (IL12R*β*2 - Clone 305719) was purchased from RnD Systems (Minneapolis, MN). ChromPure human IgG (whole molecule) were purchased from Jackson Immuno Research (West Grove, PA, U.S.A.). Quantum Simply Cellular uniform microspheres conjugated to anti-mouse IgG were purchased from Bangs Laboratories (Fishers, IN).

### Quantification of protein copy number

Fluorescence-activated cytometry was performed as described [27], [38]. Quantum Simply Cellular calibration beads that contain four Quantum Simply Cellular microsphere populations with different mouse IgG antibody binding capacities were stained with fluorophore-conjugated monoclonal antibodies specific for IL12R*β*1, IL12R*β*2, or HLA-ABC. The cells were analyzed using a FACSAria flow cytometer and FACSDiva Version 6.1.1 software (BD Biosciences). No stain controls were used as negative flow cytometry controls. Single stain controls were used to establish fluorescent compensation parameters. Cellular events were identified by forward and side scatter characteristics. On average, 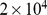 events were analyzed. Flow cytometry data was exported as FCS3.0 files and analyzed using R/Bioconductor [73].

## Supporting Information

Dataset S1 **Data.xls.** This Microsoft Excel file contains gene expression values used and scoring coefficients for each of the top 10 principal components.
(XLS)

Figure S1**Tumor characteristics associated with hierarchical clustering of patients.** Molecular pathology (top), PAM50 intrinsic tumor subtypes, posterior estimation of immune cell bias, and immune cell recruitment signature aligned to hierarchical clustering of the gene expression profiles. The molecular pathology (Normal, Ductal, Lobular, Other, ER+, PR+, HER2+) and PAM50 intrinsic tumor subtypes (Basal, HER2-like, Luminal A, Luminal B, Normal-like) are indicated by a blue vertical bar. Posterior estimation of immune cells bias is indicated by black-white shaded bar (p() = 0: black, p() = 1: white). The magnitude of the immune cell recruitment signature (Macrophages (M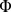), T cells, and NK cells) is indicated by a ROYGBIV color scheme, where red indicates a low average log2 median-centered value and violet indicates a high average log2 median-centered value. Dendrogram indicates the degree of similarity in gene expression among samples (columns) using the Wards minimum distance method in R. Dendrogram was calculated based on gene expression shown in Figure 1.(TIFF)

Figure S2 **Principal component analysis of gene expression values projected onto patient cohorts.** (A) Column dendrogram was calculated based on gene expression shown in Figure 1. Subtypes of invasive breast cancer cohort are indicated by color bars: group 1a - black (Normal), group 1b - blue, group 1c - green, and group 2 - red. (B) Variance captured by principal components, expressed as a percentage. (C) Within the entire population, the density distributions of subtypes, stratified by molecular pathology, marginalized along PC1 are shown for triple negative (TN - gray), HER2+ (yellow), and other subtypes (blue). Below the density distributions, the projection of invasive breast cancer cohort along PC1 and PC2 dimensions. Points are color coded as shown in panel A. Triple negative breast cancer samples in groups 1b, 1c, and 2 are filled circles. Samples derived from normal breast tissue are filled black.
(TIF)

Figure S3 **External validation of TCGA gene expression signature.** Projections along the first four principal component directions of the invasive breast cancer samples (A) and normal breast tissue samples (B) reported in four potential validation studies (black - TCGA [14], orange - Karnoub et al. [76], blue - Finak et al. [55], and red - Gluck et al. [34]). In panel B, the colored contour lines indicate the PC values that enclose 95% of the invasive breast cancer samples. Contours were estimated from the data shown in panel A by kernel density estimation. (C and D) Biplot projections of the genes along the first two principal component directions (panel C - Gluck et al. [34], panel D - TCGA [14]). Synthetic samples were generated by random bootstrap resampling with replacement of the set of all gene expression values reported for a study. The colored ovals indicate different noise thresholds by enclosing different fractions of the biplot projections of the synthetic samples (median +/−1 s.d. (red), +/−2 s.d. (yellow), +/−3 s.d. (green), +/−5 s.d. (blue), and +/−7 s.d. (violet)). (E) A biplot comparison of the covariation observed in gene expression in the Gluck study [34](blue circles) to the TCGA study [14](red circles). Projections for the same gene observed in the two different studies are connected by a line. The top 10 genes that exhibited the greatest differences between studies are highlighted in bold.
(TIF)

Figure S4 **Comparisons of gene expression using pairwise scatter plots.** (A) Genes in PC2 with high loading coefficients: *WISP1*, *GATA3*, *PPARG*, and *IL6*. (B) Comparison among GATA3 gene expression, copy number, and protein expression. Bivariate scatter plots of log2 median-centered ratios of gene expression (*GATA3*), of copy number (cnGATA3), and of protein abundance (peGATA3) as measured by reverse phase protein array. (C) Comparison among *GATA3*, *IL12RB1*, and *IL12RB2* gene expression. In all panels, the scatter plots are shown below the diagonal, marginalized histograms stratified by the two invasive breast cancer groups are shown on the diagonal, and Pearson covariation coefficients are shown above the diagonal. Results are colored by group (Breast Cancer Group 1: blue, Breast Cancer Group 2: red). All values were obtained from the TCGA website (https://tcga-data.nci.nih.gov/tcga/).
(TIF)

Figure S5 **Pairwise scatter plots for genes previously associated with tumor immunosuppression.** Genes shown include *FOXP3*, *RORC*, *GATA3*, *HMGB1* [77], *TGFB1*, *PDCD1* [78]–[80], *CD274* [78]–[80], *IL10*, *IDO1*, *ARG1*, *HIF1A* [81], *BTLA*, *HAVCR2* (TIM-3), *LAG3*, *MICA/MICB*, and *VTCN1*(B7-H4) [82]. Bivariate scatter plots of log2 median-centered ratios of gene expression are shown below the diagonal, marginalized histograms stratified by the three groups are shown on the diagonal, and correlation coefficients are shown above the diagonal. Results are colored by group (Breast Cancer Group 1: blue, Breast Cancer Group 2: red, Normal breast tissue: black).
(TIFF)

Figure S6 **Posterior estimation of immune bias using revised T helper cell polarization signatures.** Mean posterior probability associated with T helper cell and macrophage polarization in each group (Group 1 - blue, Group 2 - red, Normal - green) were estimated based upon a revised mutually exclusive gene expression signature that are associated with each cell subset, as discussed in the main text. The mean values in posterior distributions of the null hypothesis in immune bias were estimated for each of the 1000 bootstrap resamples and shown as a distribution (gray shaded density distribution).
(TIFF)

Figure S7 **Model-based inference of type 1 immune polarization is a better predictor of improved outcome than a random model of identical size.** The x-axis denotes the hazard ratio of the clinical outcome associated with either a model representing type 1 T cell polarization (red squares) or a random model assembled from a random sample of the genes listed in Table 1 (box and whisker plot) obtained using a Cox proportional hazards model for the indicated time frames (1 Year, 3 Years, 5 Years, and 6 Years). The distribution in hazard ratio associated with the random model was assembled from 1000 bootstrapped replicates (box and whisker plot), where the median is represented by the vertical bar, the first through the third quartile is indicated by the box, and the whiskers indicate ±2.7 standard deviations. Outliers are indicated by the circles and suggest that the distribution in hazard ratios are skewed towards lower hazard ratios. This is not surprising as the genes listed in Table 1 are resampled but genes involved in anti-tumor immunity are overrepresented. An important point here is that we are not regressing a random immune signature to clinical outcome within essentially the same data set but used an immune signature derived from independent studies that has strong mechanistic interpretation. Given the extensive literature describing gene signatures associated with T cell polarization, the signature has a low a priori likelihood for a Type 1 error, although for this bootstrap example we assume equal a priori likelihood for this signature as a random model. As suggested by the skewed tail, one could identify a better signature based upon correlation between clinical outcome and a model created from some permutation of the genes in Table 1. However from a mechanistic perspective, this could be interpreted as overfitting the data.
(TIF)

Table S1 **Results for Cox proportional hazards regression model.** The Cox model: (Survival∼p(Th1 T cell)+Molecular Pathology). The results suggest that the posterior estimate of a Th1 immune cell polarization gene expression signature (i.e., p(Th1 T cell)) is a predictor of overall survival independent of the molecular pathology. We also found that the other T helper cell polarization states (i.e., p(Th2 T cell), p(Th17 T cell), and p(iTreg T cell)) were not predictive.
(PDF)

Text S1 **TextS1.pdf.** This PDF file contains: 1. Principal component analysis 2. External validation of the TCGA gene expression signature References.
(PDF)
