# Supplementary figures and images for: Induction of Wnt-Inducible Signaling Protein-1 Correlates with Invasive Breast Cancer Oncogenesis and Reduced Type 1 Cell-Mediated Cytotoxic Immunity: A Retrospective Study
Source: PLoS Comput Biol. 2014 Jan 9;10(1):e1003409. doi: 10.1371/journal.pcbi.1003409 (PMC3890420; doi:10.1371/journal.pcbi.1003409)

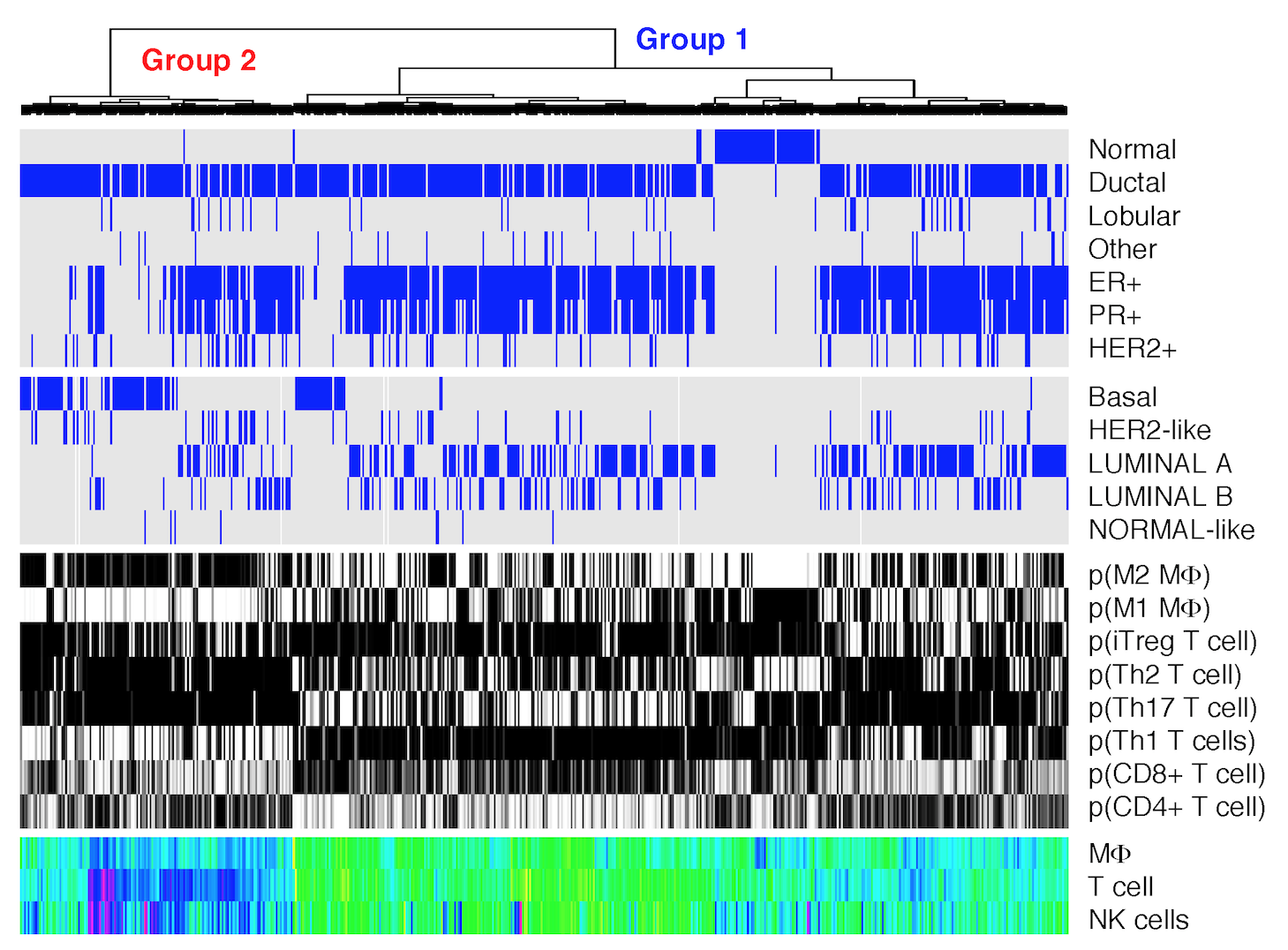

Supplement: Figure S1 — Tumor characteristics associated with hierarchical clustering of patients. Molecular pathology (top), PAM50 intrinsic tumor subtypes, posterior estimation of immune cell bias, and immune cell recruitment signature aligned to hierarchical clustering of the gene expression profiles. The molecular pathology (Normal, Ductal, Lobular, Other, ER+, PR+, HER2+) and PAM50 intrinsic tumor subtypes (Basal, HER2-like, Luminal A, Luminal B, Normal-like) are indicated by a blue vertical bar. Posterior estimation of immune cells bias is indicated by black-white shaded bar (p() = 0: black, p() = 1: white). The magnitude of the immune cell recruitment signature (Macrophages (M), T cells, and NK cells) is indicated by a ROYGBIV color scheme, where red indicates a low average log2 median-centered value and violet indicates a high average log2 median-centered value. Dendrogram indicates the degree of similarity in gene expression among samples (columns) using the Wards minimum distance method in R. Dendrogram was calculated based on gene expression shown in Figure 1. (TIFF) [file pcbi.1003409.s002.tiff]

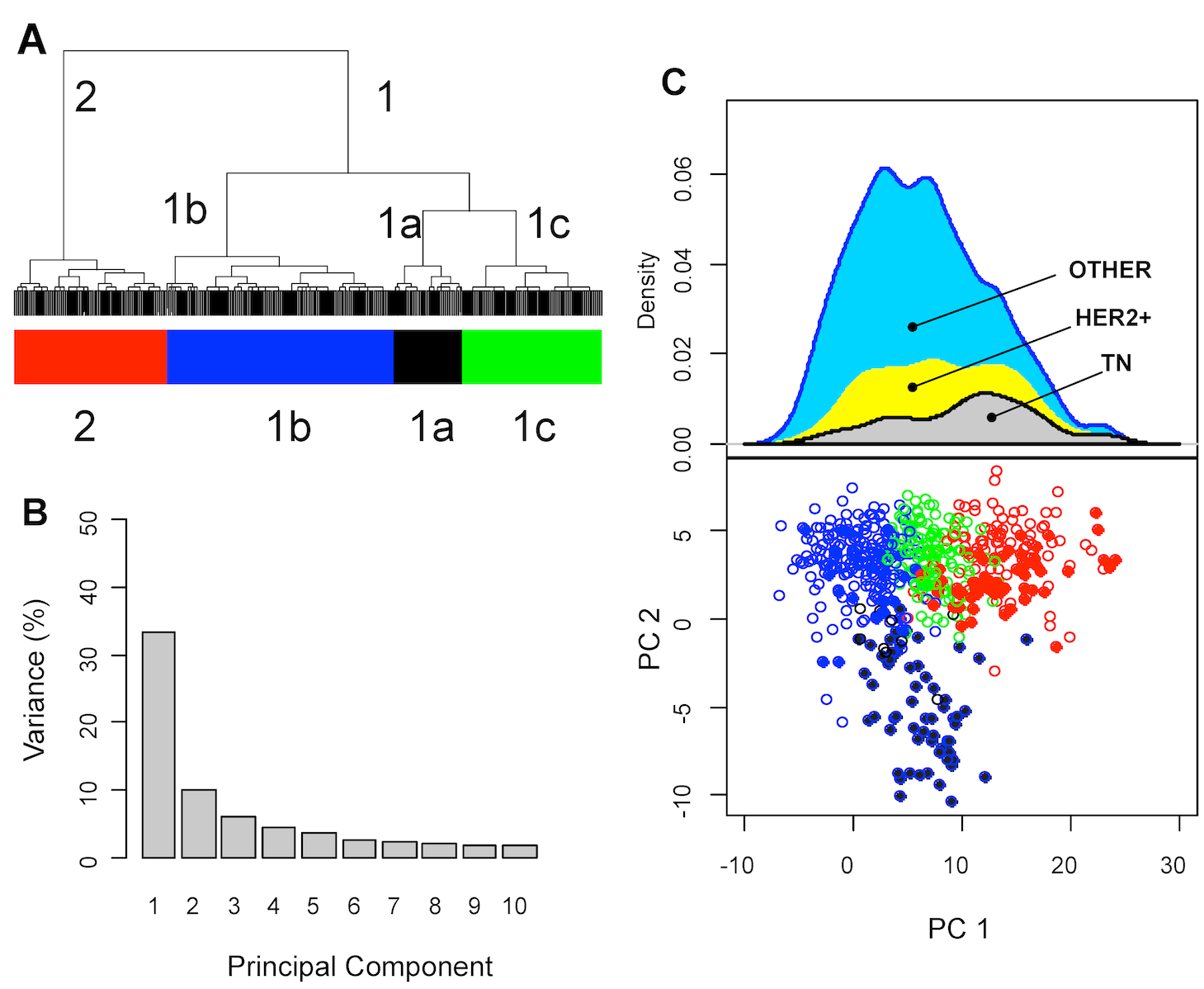

Supplement: Figure S2 — Principal component analysis of gene expression values projected onto patient cohorts. (A) Column dendrogram was calculated based on gene expression shown in Figure 1. Subtypes of invasive breast cancer cohort are indicated by color bars: group 1a - black (Normal), group 1b - blue, group 1c - green, and group 2 - red. (B) Variance captured by principal components, expressed as a percentage. (C) Within the entire population, the density distributions of subtypes, stratified by molecular pathology, marginalized along PC1 are shown for triple negative (TN - gray), HER2+ (yellow), and other subtypes (blue). Below the density distributions, the projection of invasive breast cancer cohort along PC1 and PC2 dimensions. Points are color coded as shown in panel A. Triple negative breast cancer samples in groups 1b, 1c, and 2 are filled circles. Samples derived from normal breast tissue are filled black. (TIF) [file pcbi.1003409.s003.tif]

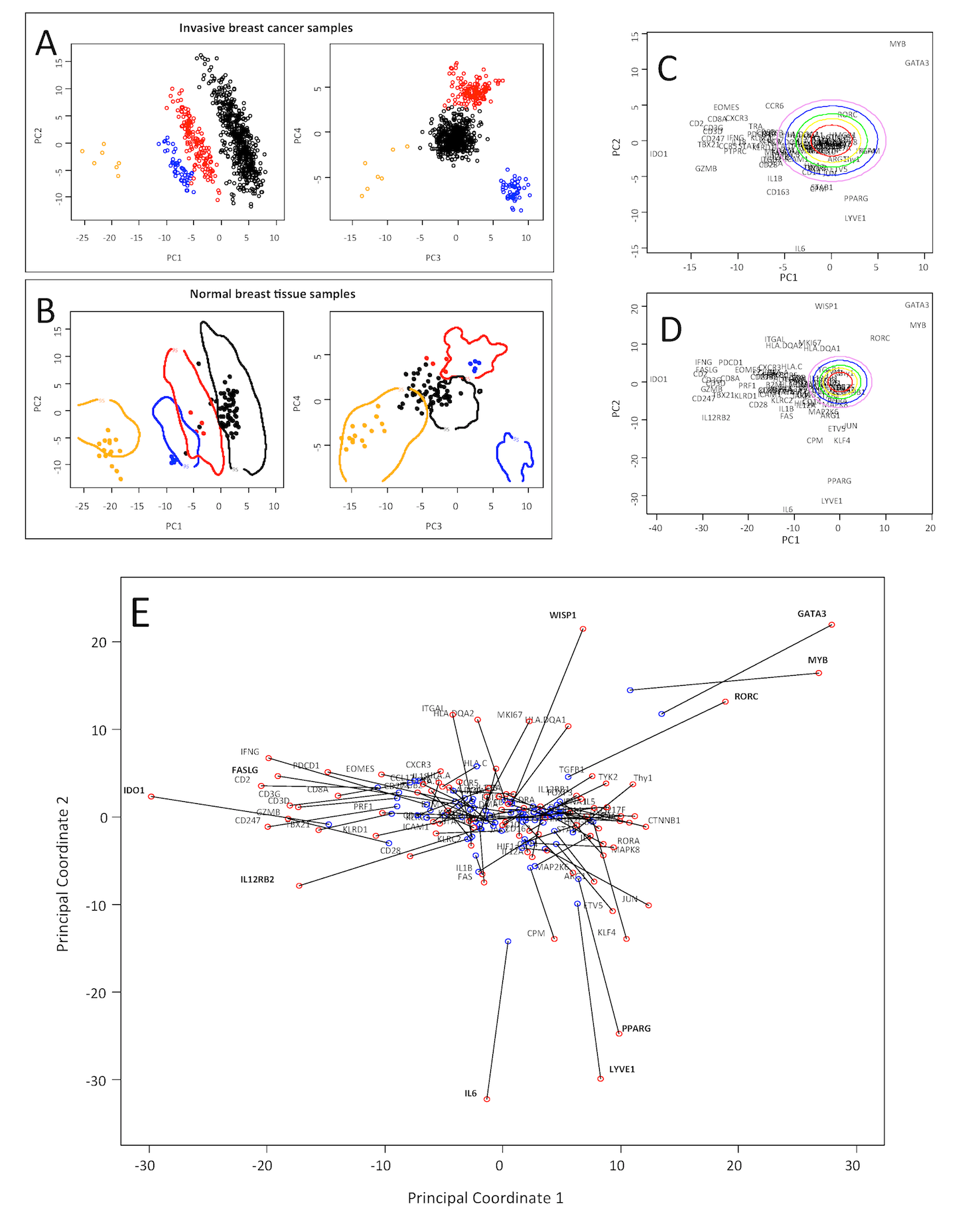

Supplement: Figure S3 — External validation of TCGA gene expression signature. Projections along the first four principal component directions of the invasive breast cancer samples (A) and normal breast tissue samples (B) reported in four potential validation studies (black - TCGA [14], orange - Karnoub et al. [76], blue - Finak et al. [55], and red - Gluck et al. [34]). In panel B, the colored contour lines indicate the PC values that enclose 95% of the invasive breast cancer samples. Contours were estimated from the data shown in panel A by kernel density estimation. (C and D) Biplot projections of the genes along the first two principal component directions (panel C - Gluck et al. [34], panel D - TCGA [14]). Synthetic samples were generated by random bootstrap resampling with replacement of the set of all gene expression values reported for a study. The colored ovals indicate different noise thresholds by enclosing different fractions of the biplot projections of the synthetic samples (median +/−1 s.d. (red), +/−2 s.d. (yellow), +/−3 s.d. (green), +/−5 s.d. (blue), and +/−7 s.d. (violet)). (E) A biplot comparison of the covariation observed in gene expression in the Gluck study [34](blue circles) to the TCGA study [14](red circles). Projections for the same gene observed in the two different studies are connected by a line. The top 10 genes that exhibited the greatest differences between studies are highlighted in bold. (TIF) [file pcbi.1003409.s004.tif]

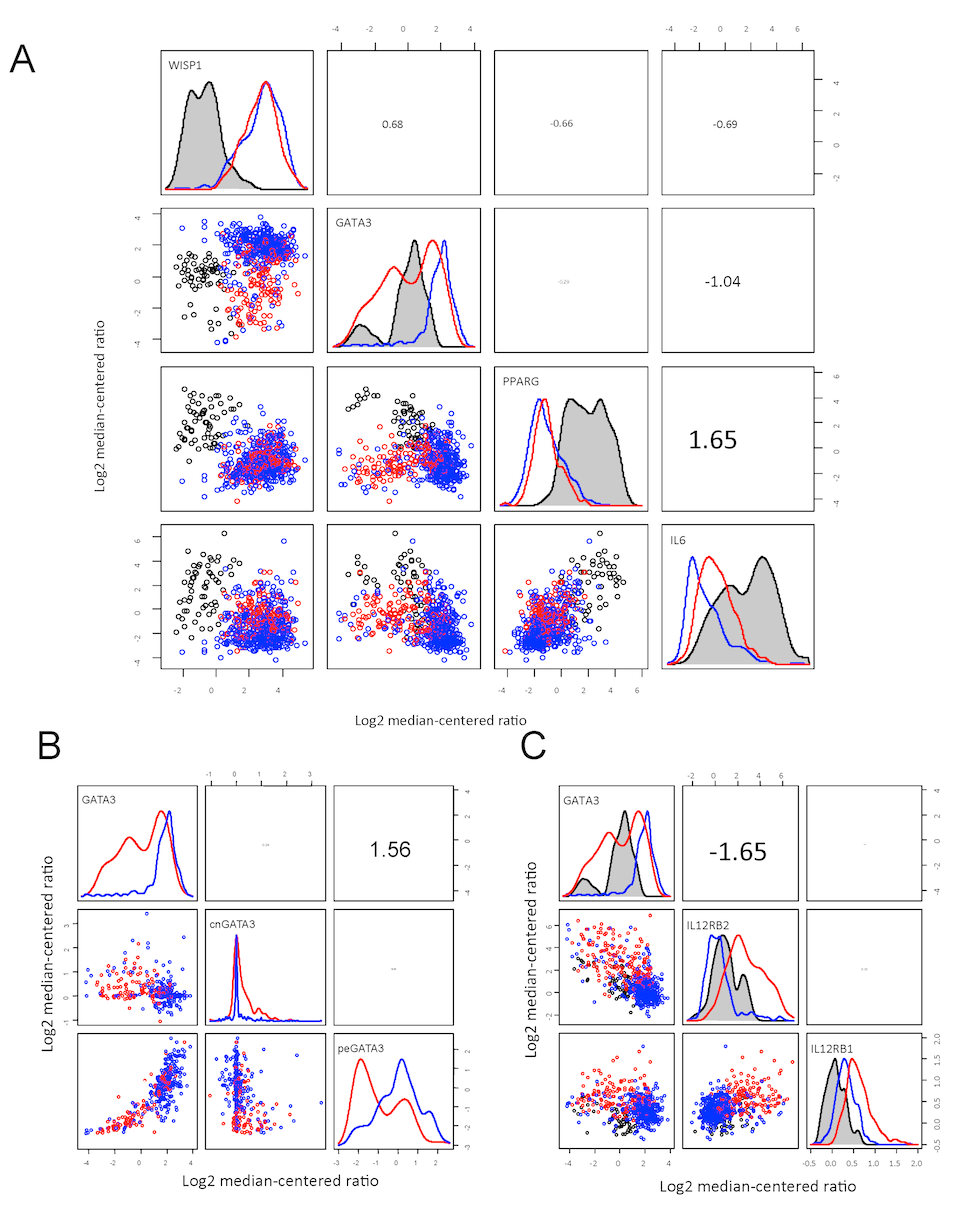

Supplement: Figure S4 — Comparisons of gene expression using pairwise scatter plots. (A) Genes in PC2 with high loading coefficients: WISP1, GATA3, PPARG, and IL6. (B) Comparison among GATA3 gene expression, copy number, and protein expression. Bivariate scatter plots of log2 median-centered ratios of gene expression (GATA3), of copy number (cnGATA3), and of protein abundance (peGATA3) as measured by reverse phase protein array. (C) Comparison among GATA3, IL12RB1, and IL12RB2 gene expression. In all panels, the scatter plots are shown below the diagonal, marginalized histograms stratified by the two invasive breast cancer groups are shown on the diagonal, and Pearson covariation coefficients are shown above the diagonal. Results are colored by group (Breast Cancer Group 1: blue, Breast Cancer Group 2: red). All values were obtained from the TCGA website (https://tcga-data.nci.nih.gov/tcga/). (TIF) [file pcbi.1003409.s005.tif]

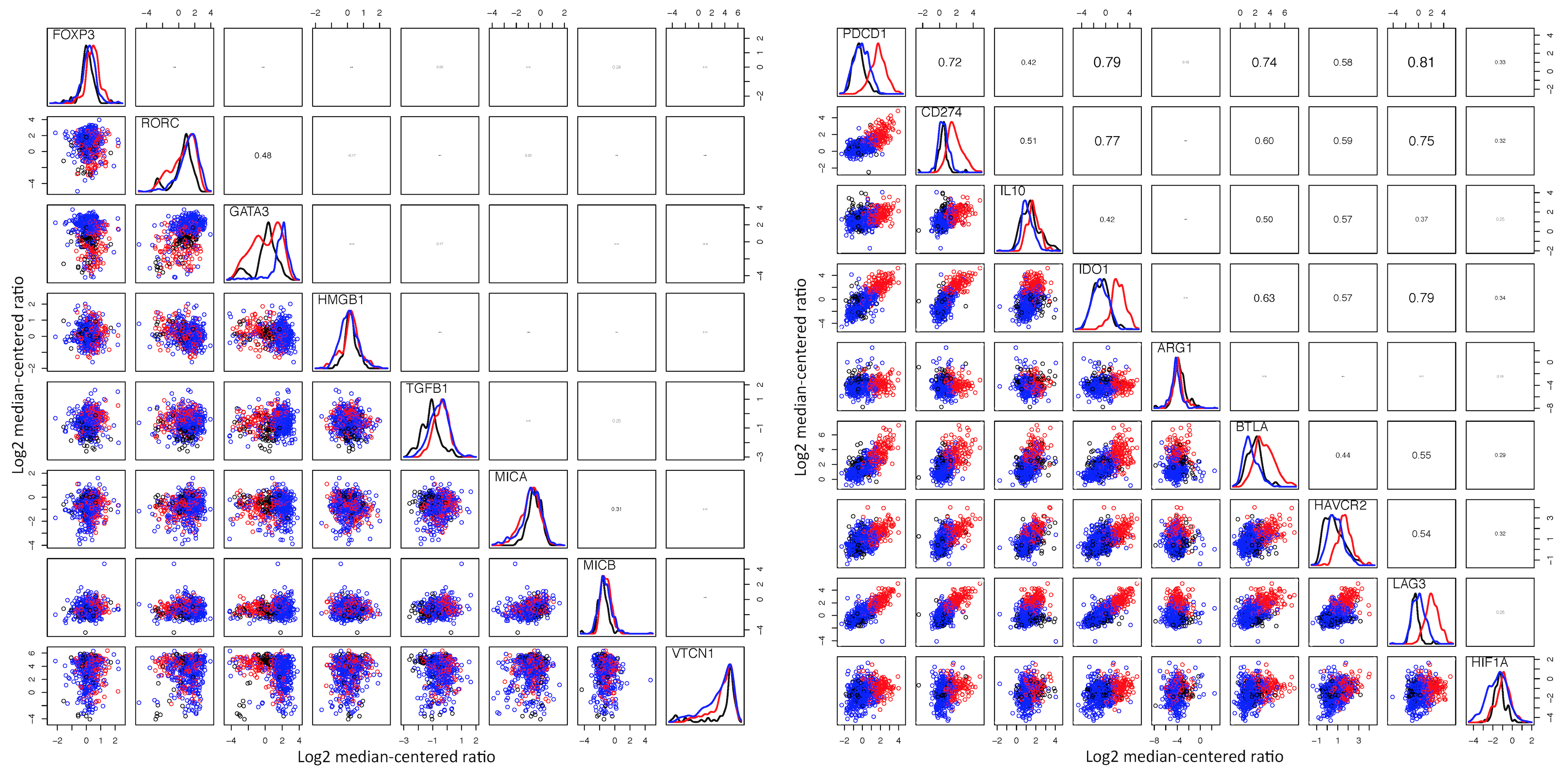

Supplement: Figure S5 — Pairwise scatter plots for genes previously associated with tumor immunosuppression. Genes shown include FOXP3, RORC, GATA3, HMGB1 [77], TGFB1, PDCD1 [78]–[80], CD274 [78]–[80], IL10, IDO1, ARG1, HIF1A [81], BTLA, HAVCR2 (TIM-3), LAG3, MICA/MICB, and VTCN1(B7-H4) [82]. Bivariate scatter plots of log2 median-centered ratios of gene expression are shown below the diagonal, marginalized histograms stratified by the three groups are shown on the diagonal, and correlation coefficients are shown above the diagonal. Results are colored by group (Breast Cancer Group 1: blue, Breast Cancer Group 2: red, Normal breast tissue: black). (TIFF) [file pcbi.1003409.s006.tiff]

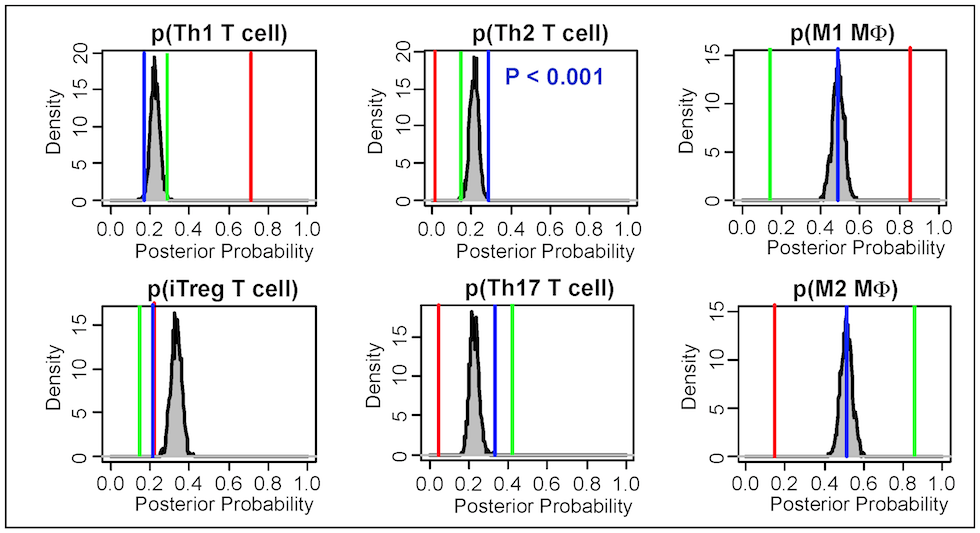

Supplement: Figure S6 — Posterior estimation of immune bias using revised T helper cell polarization signatures. Mean posterior probability associated with T helper cell and macrophage polarization in each group (Group 1 - blue, Group 2 - red, Normal - green) were estimated based upon a revised mutually exclusive gene expression signature that are associated with each cell subset, as discussed in the main text. The mean values in posterior distributions of the null hypothesis in immune bias were estimated for each of the 1000 bootstrap resamples and shown as a distribution (gray shaded density distribution). (TIFF) [file pcbi.1003409.s007.tiff]

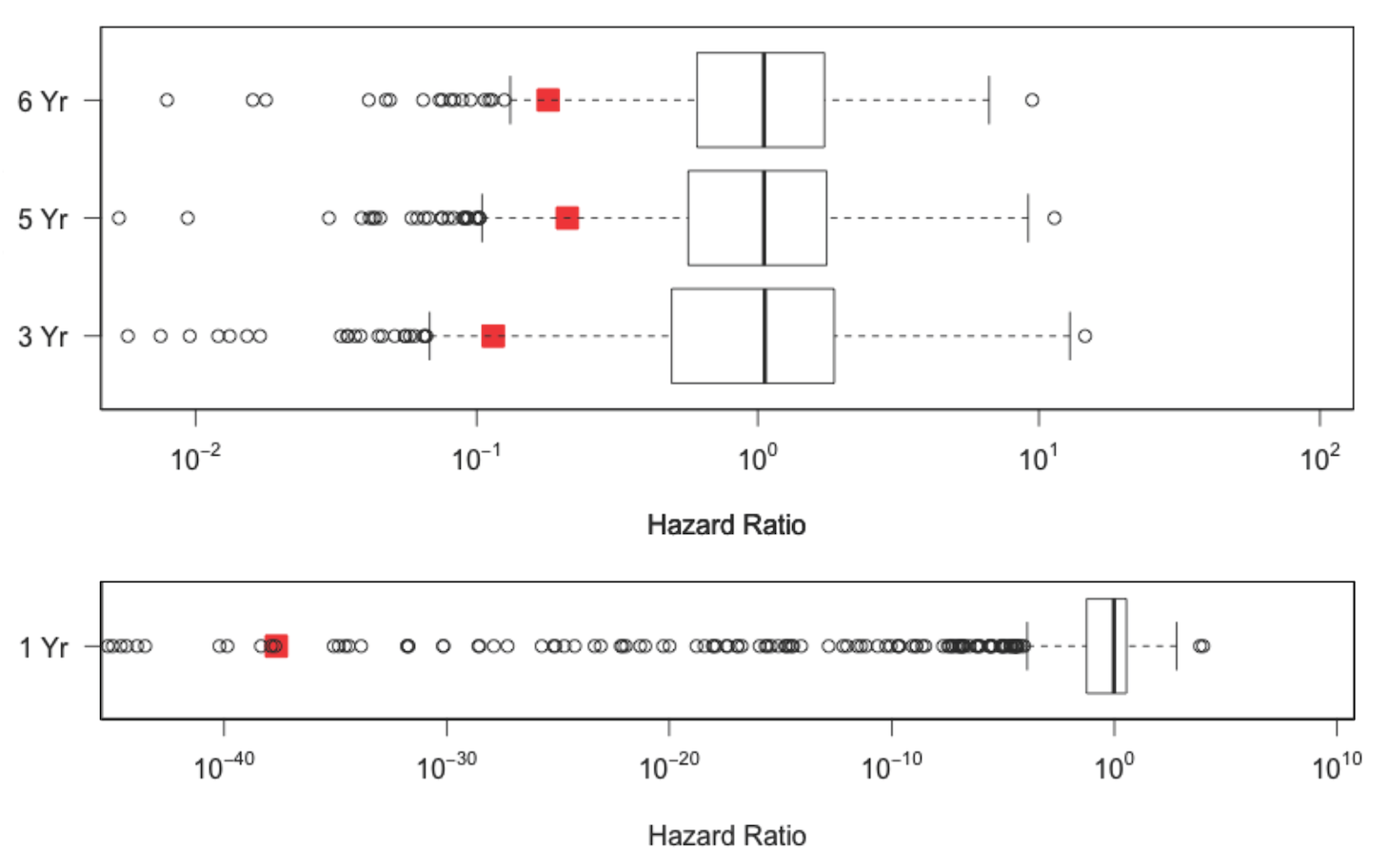

Supplement: Figure S7 — Model-based inference of type 1 immune polarization is a better predictor of improved outcome than a random model of identical size. The x-axis denotes the hazard ratio of the clinical outcome associated with either a model representing type 1 T cell polarization (red squares) or a random model assembled from a random sample of the genes listed in Table 1 (box and whisker plot) obtained using a Cox proportional hazards model for the indicated time frames (1 Year, 3 Years, 5 Years, and 6 Years). The distribution in hazard ratio associated with the random model was assembled from 1000 bootstrapped replicates (box and whisker plot), where the median is represented by the vertical bar, the first through the third quartile is indicated by the box, and the whiskers indicate ±2.7 standard deviations. Outliers are indicated by the circles and suggest that the distribution in hazard ratios are skewed towards lower hazard ratios. This is not surprising as the genes listed in Table 1 are resampled but genes involved in anti-tumor immunity are overrepresented. An important point here is that we are not regressing a random immune signature to clinical outcome within essentially the same data set but used an immune signature derived from independent studies that has strong mechanistic interpretation. Given the extensive literature describing gene signatures associated with T cell polarization, the signature has a low a priori likelihood for a Type 1 error, although for this bootstrap example we assume equal a priori likelihood for this signature as a random model. As suggested by the skewed tail, one could identify a better signature based upon correlation between clinical outcome and a model created from some permutation of the genes in Table 1. However from a mechanistic perspective, this could be interpreted as overfitting the data. (TIF) [file pcbi.1003409.s008.tif]
