# Supplementary material for: Induction of Wnt-Inducible Signaling Protein-1 Correlates with Invasive Breast Cancer Oncogenesis and Reduced Type 1 Cell-Mediated Cytotoxic Immunity: A Retrospective Study
Source: PLoS Comput Biol. 2014 Jan 9;10(1):e1003409. doi: 10.1371/journal.pcbi.1003409 (PMC3890420; doi:10.1371/journal.pcbi.1003409)
Supplement: Table S1 — Results for Cox proportional hazards regression model. The Cox model: (Survival∼p(Th1 T cell)+Molecular Pathology). The results suggest that the posterior estimate of a Th1 immune cell polarization gene expression signature (i.e., p(Th1 T cell)) is a predictor of overall survival independent of the molecular pathology. We also found that the other T helper cell polarization states (i.e., p(Th2 T cell), p(Th17 T cell), and p(iTreg T cell)) were not predictive. (PDF) [file pcbi.1003409.s009.pdf]

Table S1 - Results for Cox proportional hazards regression model.

The Cox model: (Survival $\sim$ p(Th1 T cell) + Molecular Pathology). The results suggest that the posterior estimate of a Th1 immune cell polarization gene expression signature (i.e., p(Th1 T cell)) is a predictor of overall survival independent of the molecular pathology. We also found that the other T helper cell polarization states (i.e., p(Th2 T cell), p(Th17 T cell), and p(iTreg T cell)) were not predictive.

Survival	p(Th1 T cell)			Molecular Pathology		
(Years)	p-value	Hazard ratio	95% C.I.	p-value	Hazard ratio	95% C.I.
1	0.001	< 1e-15	1e-61 – 1e-15	0.108	5.41	0.69 – 42.26
3	0.035	0.115	0.015 – 0.859	0.019	2.064	1.128 – 3.78
5	0.062	0.210	0.041 – 1.083	0.020	1.971	1.115 – 3.49
6	0.034	0.180	0.037 – 0.875	0.013	1.938	1.152 – 3.26

Molecular Pathology defined as TN = 3, HER2+ = 2, ER+/PR+ = 1.
