# Supplementary material for: Induction of Wnt-Inducible Signaling Protein-1 Correlates with Invasive Breast Cancer Oncogenesis and Reduced Type 1 Cell-Mediated Cytotoxic Immunity: A Retrospective Study
Source: PLoS Comput Biol. 2014 Jan 9;10(1):e1003409. doi: 10.1371/journal.pcbi.1003409 (PMC3890420; doi:10.1371/journal.pcbi.1003409)
Supplement: Text S1 — TextS1.pdf. This PDF file contains: 1. Principal component analysis 2. External validation of the TCGA gene expression signature References. (PDF) [file pcbi.1003409.s010.pdf]

Text Supplement for "Induction of Wnt-inducible signaling protein-1 correlates with invasive breast cancer transformation and reduced type 1 cell-mediated cytotoxic immunity: a retrospective study"

David J. Klinke II[1,2]
[1]Department of Chemical Engineering and Mary Babb Randolph Cancer Center
West Virginia University, Morgantown, WV 25606 USA
[2]Department of Microbiology, Immunology, and Cell Biology
West Virginia University, Morgantown, WV 25606 USA

November 21, 2013

Contact Info:		Department of Chemical Engineering
E-mail:	david.klinke@mail.wvu.edu	West Virginia University
Phone:	(304)293-9346	P.O. Box 6102
Fax:	(304)293-4139	Morgantown, WV 26506-6102

This PDF file includes:

1. Principal component analysis
2. External validation of the TCGA gene expression signature

1 Principal component analysis

To aid in the biological interpretation of the cohorts identified by hierarchical clustering, the variation and correlation among the gene expression measurements were characterized using principal component analysis (PCA). PCA is a multivariate statistical technique that allows for the discovery of variables that form coherent subsets and that vary independently from other subsets of variables. All of the variables in a particular subset are combined into components. PCA enables creating a lower-dimensional linear description of the population. The linear relationship is shown in Equation 1, where v's are gene expression values for the p patient and C's are the scoring coefficients for the ith principal component (PC) and n^{th} gene expression variable.

$$PC_{i,p} = C1_i \cdot v1_p + C2_i \cdot v2_p + \ldots + Cn_i \cdot vn_p \tag{1}$$

The scoring coefficients for each of the top 10 principal components are listed in Supplemental Table 1. The scoring coefficient quantify the relative weight of a variable in a principal component such that a value for the GATA3 scoring coefficient of 0.4214 in PC3 means that 17.8% (100×0.4214^2) of the variance in GATA3 expression is captured in Principal Component 3.

2 External validation of the TCGA gene expression signature

While retrospective analysis of the TCGA study aimed to validate the in vitro results that identified WISP1 as a paracrine inhibitor of IL-12 bioactivity, we also compared the immune signature observed in the TCGA data against other microarray-based gene expression studies in invasive breast cancer. We selected three recent studies on invasive breast cancer that also included normal tissue samples ([GEO:GSE9014][1], [GEO:GSE22358][2], and [GEO:GSE8977][3]). However, comparing gene expression values across studies presents challenges. Subtle differences in sample processing, batch effects during microarray fabrication, or design of probe sequences introduce systematic biases in the gene expression profiles. While many algorithms have been developed to identify and remove such batch effects [4, 5, 6], the underlying assumption in applying these algorithms to merge different gene expression values is that the microarray results represent samples of the same distribution in biological states [7]. The number and diversity of the samples contained within the invasive breast cancer TCGA data set presents a challenge for identifying a comparable validation study. To compare the gene expression signatures among these four studies, we performed principal component analysis on the collective data set (see Figure S3). Significant principal components - that is the data set contains information to inform a principal component - were identified as those that had eigenvalues above a non-informed threshold. The threshold values were estimated by bootstrapping ($n_{boot} = 500$) - that is PCA was performed on a synthetic data set ($n_{sample} = 250$) that was obtained by random sampling with replacement from the set of all observed gene expression values. The first four principal components were above the non-informed threshold and captured 58% of the variance in the collective data

set. As shown in Panels A and B in Figure S3, projections of the invasive breast cancer and normal breast tissue samples stratified by study along these four principal components graphically summarized the differences among these studies.

In comparing results obtained from invasive breast cancer versus the normal breast tissue samples, gene expression values from Gluck et al. exhibited the most similarity to the TCGA results. The other two studies reported gene expression values derived from stromal cells isolated using laser capture microdissection in Finak et al. or using antibody-based cell sorting in Karnoub et al. from primary breast tumors and normal breast tissue. In contrast, Gluck and coworkers performed gene expression analysis on core biopsy specimens obtained prior to treatment, similar to the TCGA study design. While the gene expression data reported by Gluck et al. were used for subsequent analysis, the distribution in patient population characteristics and low number of normal breast tissue samples (4) preclude a direct validation of an immune signature correlate of overall survival. Instead, we determined the similarity in the immune gene signatures between these two studies.

Principal component analysis can be applied to the patient samples to identify patient samples that express similar unique patterns of gene expression, as described in the main text. Each unique pattern is associated with a single independent principal component. Alternatively, PCA can be applied to identify genes that co-vary, as shown in Figure S3 Panels C-E and referred to as principal coordinate analysis. We used bootstrap resampling ($n_{boot} = 500$) to establish a threshold below which gene covariation may be due to random chance - that is a noise threshold, as indicated by the colored ovals in Figure S3 Panels C and D. Collectively, the distribution in covariation of gene expression relative to the noise threshold provides an estimate of the information contained within the gene expression study. As principal coordinates are independent, the projection of gene along the corresponding axis indicates the degree to which the expression of two genes are related and the distance from the origin indicates the strength of the covariation within the data set. For instance, *IFNG*, *FASLG*, *CD3G*, *CD2*, *GZMB*, *TBX21*, *CD8A*, *PRF1*, and *EOMES* are all aligned along the negative PC1 direction. Biologically, the similar location of these genes is expected as these genes are all associated with type 1 cell-mediated immunity. In contrast, *IL6*, *LYVE1*, and *PPARG* are all aligned along the negative PC2 direction. This suggests that the strength of covariation among these genes is high and that these genes vary independently from the genes associated with type 1 cell-mediated immunity. As the biplot projections of more genes in the Gluck study are located within the noise threshold relative to the TCGA study (49 genes in the Gluck study versus 25 genes in the TCGA study are located within 7 standard deviations from the origin, as indicated by the violet oval), the biplot projections imply that there is more information about the covariation of gene expression contained within the TCGA study.

We assessed the differences in gene covariation inferred from each study by comparing the biplot projections. As shown in Figure S3E, the covariation in gene expression was similar between the two studies as the line segments mainly extend in the radial direction and exhibited a greater magnitude in the TCGA study as the corresponding points were located farther from the origin. The top ten genes with the greatest difference between the Gluck and TCGA data sets were, in order of difference, *WISP1*, *FASLG*, *LYVE1*, *IL6*, *PPARG*, *GATA3*, *IL12RB2*, *MYB*, *RORC*, and *IDO1*. The differences along principal coordinate axis 2 are mainly attributed to the additional normal breast tissue samples included in the TCGA

study. Collectively, the results suggest that *GATA3* expression varies both inversely with the genes associated with type 1 cell-mediated immunity and directly with the expression of genes associated with oncogenesis, namely *WISP1*.

References

[1] Finak G, Bertos N, Pepin F, Sadekova S, Souleimanova M, et al. (2008) Stromal gene expression predicts clinical outcome in breast cancer. Nat Med 14: 518–527.

[2] Gluck S, Ross JS, Royce M, McKenna EF, Perou CM, et al. (2012) TP53 genomics predict higher clinical and pathologic tumor response in operable early-stage breast cancer treated with docetaxel-capecitabine ± trastuzumab. Breast Cancer Res Treat 132: 781–791.

[3] Karnoub AE, Dash AB, Vo AP, Sullivan A, Brooks MW, et al. (2007) Mesenchymal stem cells within tumour stroma promote breast cancer metastasis. Nature 449: 557–563.

[4] Benito M, Parker J, Du Q, Wu J, Xiang D, et al. (2004) Adjustment of systematic microarray data biases. Bioinformatics 20: 105–114.

[5] Johnson WE, Li C, Rabinovic A (2007) Adjusting batch effects in microarray expression data using empirical Bayes methods. Biostatistics 8: 118–127.

[6] Taminau J, Meganck S, Lazar C, Steenhoff D, Coletta A, et al. (2012) Unlocking the potential of publicly available microarray data using inSilicoDb and inSilicoMerging R/Bioconductor packages. BMC Bioinformatics 13: 335.

[7] Sims AH, Smethurst GJ, Hey Y, Okoniewski MJ, Pepper SD, et al. (2008) The removal of multiplicative, systematic bias allows integration of breast cancer gene expression datasets - improving meta-analysis and prediction of prognosis. BMC Med Genomics 1: 42.
